# Constraint-based modeling of bioenergetic differences between synaptic and non-synaptic components of dopaminergic neurons in Parkinson’s disease

**DOI:** 10.3389/fncom.2025.1594330

**Published:** 2025-06-05

**Authors:** Xi Luo, Diana C. El Assal, Yanjun Liu, Samira Ranjbar, Ronan M.T. Fleming

**Affiliations:** ^1^School of Medicine, University of Galway, Galway, Ireland; ^2^Division of Science and Math, New York University Abu Dhabi, Abu Dhabi, United Arab Emirates; ^3^Division of Systems Biomedicine and Pharmacology, Leiden Academic Center for Drug Research, Leiden University, Leiden, Netherlands

**Keywords:** Parkinson’s disease, bioenergetics, synaptic, non-synaptic, modeling

## Abstract

**Introduction:**

Emerging evidence suggests that different metabolic characteristics, particularly bioenergetic differences, between the synaptic terminal and soma may contribute to the selective vulnerability of dopaminergic neurons in patients with Parkinson’s disease (PD).

**Method:**

To investigate the metabolic differences, we generated four thermodynamically flux-consistent metabolic models representing the synaptic and non-synaptic (somatic) components under both control and PD conditions. Differences in bioenergetic features and metabolite exchanges were analyzed between these models to explore potential mechanisms underlying the selective vulnerability of dopaminergic neurons. Bioenergetic rescue analyses were performed to identify potential therapeutic targets for mitigating observed energy failure and metabolic dysfunction in PD models.

**Results:**

All models predicted that oxidative phosphorylation plays a significant role under lower energy demand, while glycolysis predominates when energy demand exceeds mitochondrial constraints. The synaptic PD model predicted a lower mitochondrial energy contribution and higher sensitivity to Complex I inhibition compared to the non-synaptic PD model. Both PD models predicted reduced uptake of lysine and lactate, indicating coordinated metabolic processes between these components. In contrast, decreased methionine and urea uptake was exclusively predicted in the synaptic PD model, while decreased histidine and glyceric acid uptake was exclusive to the non-synaptic PD model. Furthermore, increased flux of the mitochondrial ornithine transaminase reaction (ORNTArm), which converts oxoglutaric acid and ornithine into glutamate-5-semialdehyde and glutamate, was predicted to rescue bioenergetic failure and improve metabolite exchanges for both the synaptic and non-synaptic PD models.

**Discussion:**

The predicted differences in ATP contribution between models highlight the bioenergetic differences between these neuronal components, thereby contributing to the selective vulnerability observed in PD. The observed differences in metabolite exchanges reflect distinct metabolic patterns between these neuronal components. Additionally, mitochondrial ornithine transaminase was predicted to be the potential bioenergetic rescue target for both the synaptic and non-synaptic PD models. Further research is needed to validate these dysfunction mechanisms across different components of dopaminergic neurons and to explore targeted therapeutic strategies for PD patients.

## 1 Introduction

Parkinson’s disease (PD) is an age-related, progressive neurodegenerative disorder characterized by the selective vulnerability of dopaminergic neurons in the substantia nigra pars compacta (SNpc) (Gonzalez-Rodriguez et al., 2020; Ramesh and Arachchige, 2023). Neurons in the substantia nigra project axons to two regions of the dorsal striatum, the caudate nucleus and the putamen, forming the well-known nigrostriatal pathway (Pissadaki and Bolam, 2013). This pathway is essential for fine motor skills and their functions, significantly contributing to the motor symptoms of PD. Recent studies have demonstrated that neurodegeneration in PD follows a retrograde progression, which highlights distinct dysfunctions between the synaptic terminals and the neuronal soma of dopaminergic neurons (Burke and O’Malley, 2013; Morales et al., 2021; Calabresi et al., 2023). Several factors, such as variations in oxidative stress, differences in calcium distribution, and metabolomic diversity, have been identified that may contribute to the selective vulnerability of dopaminergic neurons in the SNpc (Jové et al., 2014; Gonzalez-Rodriguez et al., 2020; Watanabe et al., 2024). In particular, metabolic differences, especially in mitochondrial morphology and bioenergetics between the synaptic terminal and the soma, may play a critical role in this selective vulnerability (Brown et al., 2006; Stauch et al., 2014; Zanfardino et al., 2021; Faria-Pereira and Morais, 2022). This may further result in dynamic metabolomic patterns in PD, contributing to the heterogeneity observed in metabolomic studies of PD patients (Luo et al., 2024).

Mitochondria are highly dynamic organelles with a complex organization that enables them to perform essential biological functions such as regulating lipid and amino acid metabolism (Todkar et al., 2017), as well as maintaining calcium homeostasis (Borsche et al., 2021; Walkon et al., 2022). Abnormal mitochondrial function in PD patients, characterized by significantly reduced Complex I activity, has been observed since 1990 (Schapira et al., 1990). Due to their long, unmyelinated axons and numerous dopamine release sites, dopaminergic neurons in the SNpc require substantial energy for action potential propagation, recovery, and neurotransmitter release, making them particularly vulnerable to impairments in energy metabolism (Pissadaki and Bolam, 2013; Cunnane et al., 2020). Moreover, gray matter regions, including the substantia nigra and dorsal striatum, consume more energy than the brain average (Attwell and Laughlin, 2001; Tomasi et al., 2013). Consequently, disruptions in energy metabolism can lead to a balance between energy supply and demand, impairing energy-intensive cellular processes.

Mitochondrial morphology and size are regulated by fission and fusion processes, which are crucial for maintaining their integrity, distribution, and bioenergetic function (Cheng et al., 2022). Typically, the majority of neuronal mitochondria are located in synapses, where they support vesicular neurotransmitter release and synaptic vesicle cycling (Palikaras and Tavernarakis, 2020). Fission process in the soma enables mitochondrial transport into axon terminals, while fusion process helps reduce the burden on stressed mitochondria (Rambold et al., 2011; Berthet et al., 2014). Glycolysis, although less efficient, rapidly generate ATP to meet energy demands for neuronal development. In contrast, oxidative phosphorylation is highly efficient and generates the highest ATP levels in differentiated neurons (Rumpf et al., 2023). The relative contributions of glycolysis and oxidative phosphorylation remain unclear, partly due to varying energy demands under different functional states. Glycolytic ATP may be especially important for action potential propagation and recovery in dendrites, the soma, and axons during the resting state (Gruetter et al., 2001; Faria-Pereira and Morais, 2022; Li et al., 2023), while oxidative phosphorylation underpins the primary mechanisms of brain information processing (Hall et al., 2012; Rumpf et al., 2023). A deeper understanding of the bioenergetic differences between the synaptic and non-synaptic components is therefore essential.

To comprehensively understand the metabolic changes in both physiological and pathological states, constraint-based reconstruction and analysis (COBRA) has been widely used to build cell type-specific and condition-specific metabolic models (Heirendt et al., 2019). Since the development of a metabolic model for central brain energy metabolism (Cakir et al., 2007), several models have been updated to enhance the understanding of brain molecular mechanisms (Büchel et al., 2013; Sertbaş et al., 2014; Özcan and Çakır, 2018). However, these brain models fail to accurately predict the metabolism of dopaminergic neurons due to the inclusion of stoichiometrically balanced flux cycles that violate the second law of thermodynamics (Schellenberger et al., 2011; Fleming et al., 2012). To address the issue, XomicsToModel, a pipeline that generates context-specific, thermodynamically flux-consistent models from global metabolic networks and omics data (Preciat et al., 2021b), has been applied to generate the metabolic models of midbrain-specific dopaminergic neurons (iDopaNeuro models) (Preciat et al., 2021a). Although the iDopaNeuro models compared well with experimental data, they offer limited insights into the distinct synaptic and somal components of dopaminergic neurons. Consequently, it is necessary to explore metabolic differences, particularly regarding bioenergetic differences, between the synaptic and non-synaptic (somal) components of dopaminergic neurons using constraint-based metabolic modeling.

To investigate metabolic differences between the synaptic and non-synaptic (somal) components of dopaminergic neurons under physiological (control) and pathological (PD) conditions, we generated four constraint-based metabolic models from the global human metabolic model (Recon3D Model) (Brunk et al., 2018) using “XomicsToModel” pipeline. These models were analyzed to compare the differences in bioenergetic features and metabolite exchanges, thereby exploring potential mechanisms underlying the selective vulnerability of dopaminergic neurons. First, we conducted bioenergetic analyses under varying energy demands to evaluate ATP contributions among the models. Subsequently, we assessed sensitivity to Complex I inhibition by simulating varying levels of inhibition in each model. Next, we compared the metabolite exchanges in the synaptic and non-synaptic PD models with significantly altered cerebrospinal fluid (CSF) metabolites in PD patients (Luo et al., 2024), aiming to identify metabolic changes that are either consistent or inconsistent with PD patients. Finally, bioenergetic rescue analyses were performed to identify potential therapeutic targets for mitigating observed energy failure and metabolic dysfunction in PD models, offering insights into treatment strategies for PD patients.

## 2 Materials and methods

### 2.1 Model generation

An established pipeline, XomicsToModel, was used to generate thermodynamically flux-consistent, context-specific models (Preciat et al., 2021b) for the synaptic and non-synaptic components under control and PD status, respectively. This pipeline facilitates the integration of omics data (including genomics, transcriptomics, proteomics, metabolomics, and bibliomics) and enables the extraction of a physicochemically consistent mechanistic model from a global metabolic network, as previously demonstrated for in vitro dopaminergic neurons (Preciat et al., 2021a). In this study, we used bibliomics and transcriptomics data as input, with the human metabolism model (Recon3D Model) (Brunk et al., 2018) serving as the global metabolic network for model generation. The bibliomics data were manually curated from published neurobiochemical literature and brain metabolism models, with a particular focus on dopaminergic neuronal (iDopaNeuro) models, to improve the simulation of both synaptic and non-synaptic components of dopaminergic neurons. Transcriptomics data refer to single-cell RNA sequencing (ScRNA-seq) data obtained from a public database for dopamine neurons, enabling a more precise distinction between control and PD conditions (Kamath et al., 2022).

#### 2.1.1 Bibliomics data

Manual curation of bibliomics data prioritized central carbon metabolism and established metabolic pathways, for example, dopamine metabolism and mitochondria-associated pathways. The primary evidence in the literature is ideally derived from neurobiochemical experiments conducted using human tissue. In cases where human-specific data are unavailable, evidence from mammalian studies can be considered a suitable alternative. To satisfy the input data requirements of the XomicsToModel pipeline, bibliomics data were organized into a standardized format (Preciat et al., 2021b). The bibliomics data corresponding to each model are provided in Supplementary Files 1-4, respectively. Each file contained multiple sheets categorizing the data into the following: active genes (activeGenes), inactive genes (inactiveGenes); active reactions (activeReactions); inactive reactions (rxns2remove); new reactions to be added (rxns2add); coupled reactions representing potential degradation pathways for the same biomass precursor (coupledRxns); essential amino acids (essentialAA); uptake reactions indicating specific metabolites from the system (mediaData); and context-specific constraints (rxns2constraints).

##### 2.1.1.1 Identification of active and inactive genes

The enzymes expressed in all neurons, such as those involved in central carbon metabolism, lipid metabolism, amino acid metabolism, basic mitochondrial functions, and necessary transporters, were designated as active genes for both synaptic and non-synaptic models (Cakir et al., 2007; Panov et al., 2014; Zheng et al., 2016). Additionally, genes exhibiting high expression levels in neurons during experiments were classified as active genes (Thiele and Palsson, 2010). For example, the cytochrome P450 side-chain cleavage genes (P450scc) and the 17alpha-hydroxylase/C17-20-lyase genes (P450c17) were detected in neurons using reverse transcription polymerase chain reaction (Zwain and Yen, 1999). Furthermore, tyrosine hydroxylase was found to be co-expressed with FOXA2, NURR1, GIRK2, and VMAT2, which serve as markers of midbrain dopaminergic neurons (Schöndorf et al., 2014). Thus, these genes were designated as active in all the models. Moreover, several genes showed differential expression between the synaptic and non-synaptic components across multiple pathways, including oxidative phosphorylation, mitochondrial fission and fusion processes, and mitochondrial DNA replication and maintenance (Stauch et al., 2014; Petersen et al., 2019), which could help differentiate the synaptic and non-synaptic components of dopaminergic neurons. Accordingly, genes exhibiting significant changes in the synaptic component were designated as active in the synaptic but inactive in the non-synaptic model. Conversely, genes showing significant changes in the non-synaptic components were considered active only in the non-synaptic model. For example, most rat dopamine neurons in culture expressed VGLUT2, one of the three vesicular glutamate transporters. Thus, the VGLUT2 gene was considered active and specific to the synaptic model, while inactive in the non-synaptic model (Dal Bo et al., 2004).

Moreover, the human proteome map (Uhlén et al., 2015), constructed using an integrated omics approach and covering 32 different tissues and organs, was used to refine the active and inactive gene lists. Genes absent from the brain in the proteome map were assigned to the inactive gene list. As the brain comprises a mixture of glial cells (such as astrocytes, microglia, and oligodendrocytes) and various types of neurons, genes that were highly expressed in glial cells or show little to no expression in neurons were assigned to the inactive gene list for all components of dopaminergic neurons. For example, evidence indicates that glycogen-related enzymes are expressed at low levels in the brain (Fagerberg et al., 2014). Brain glycogen enzymes, such as glycogen branching enzyme and glycogen phosphorylase, are localized exclusively in astrocytes, where they serve as an energy reserve (Benarroch, 2005; Schönfeld and Reiser, 2013). Additionally, astrocytes are the brain’s primary steroidogenic cells, exhibiting the highest activity of corresponding genes, such as those in the 17beta-hydroxysteroid dehydrogenase (17betaHSD) family (Zwain and Yen, 1999). The GLUT5 gene, a fructose transporter responsible for fructose uptake, was identified as localized in microglia within the central nervous system (Mizuno et al., 2021). Furthermore, the transcriptional regulation of acyl-CoA synthetase and acyl-CoA thioesterase enzymes appears very low in the brains of male mice (Ellis et al., 2015). Therefore, these genes were assigned to the inactive gene list for all models. All the active and inactive genes were specified in terms of Entrez identifiers. The identifiers for genes, metabolites, and reactions were specified using the Virtual Metabolic Human (VMH) namespace^1^, a metabolic knowledge database that annotates these entities in metabolic models (Noronha et al., 2019).

##### 2.1.1.2 Identification of active and inactive metabolic reactions

Active and inactive reactions of the synaptic and non-synaptic components were primarily classified according to the activity status of their corresponding genes. When evidence of enzyme activity supporting a specific reaction in a given cell type was found, the reaction was classified as active. For example, reactions involved in glycolysis, oxidative phosphorylation, and dopamine metabolism were classified as active due to the associated genes were designated as active. When uncertainty existed regarding the classification of several possible metabolic reactions, only the related gene was added to the active list rather than each individual reaction.

In addition to gene-based classifications, several reactions were classified as active if supported by literature evidence. For instance, folate serves as a cofactor in one-carbon metabolism and DNA repair, promoting the remethylation of homocysteine (Balashova et al., 2018). Folate deficiency and elevated homocysteine levels have been linked to neurodegenerative disorders, such as Alzheimer’s disease and Parkinson’s disease, in adults and older individuals. In the brain, folate primarily exists in a polyglutamated form, with one to seven terminal gamma-linked glutamates. The penta- and hexa-derivatives are the most commonly occurring polyglutamated forms of folate in the brain (Priest et al., 1982). Therefore, the folate uptake reaction and the associated metabolic reactions involving longer polyglutamate chains were classified as active.

Additionally, new reactions from the iDopaNeuro models were included to further enhance the bibliomics data, such as the demand reactions for cardiolipin (Martinez-Vicente, 2017) and several reactions related to aminoacylase 1 (Preciat et al., 2021a). Since fully differentiated dopaminergic neurons cannot replicate, turnover constraints for their constituent biomass precursors were applied to maintain basic neuronal requirements (Preciat et al., 2021a). Consequently, all existing biomass reactions in the human metabolic model (Recon3D Model) were set as inactive reactions to avoid duplicate uptake. Additionally, if no evidence of metabolite accumulation was found, any existing demand and sink reactions in the Recon3D Model were also set as inactive.

##### 2.1.1.3 Adding specific constraints for synaptic and non-synaptic models

To more accurately simulate the molecular conversion and metabolism in the synaptic and non-synaptic components of dopaminergic neurons, we introduced specific constraints for each model. In this study, we derived quantitative constraints on biochemical reactions by referencing established models (Lewis et al., 2010; Preciat et al., 2021a) and through manual curation of neurobiochemical literature.

**General constraints for all models.** Because of the limited experimental evidence on synaptic and non-synaptic components, most literature-derived constraints are identical to those used in established neuronal models, particularly the iDopaNeuro models. Due to inconsistencies in the units used across different experimental techniques, all reaction flux constraints were converted to a uniform unit (mol/gDW/h). Here, gDW refers to the gram per dry weight of general gray matter tissue, assuming an average water content of 82.3% in human cerebral cortex gray matter (O’Brien and Sampson, 1965; Preciat et al., 2021a).

Since fully differentiated dopamine neurons cannot replicate, fulfilling the turnover demand for most cellular constituents is sufficient. Turnover constraints for dopaminergic neurons were applied to both synaptic and non-synaptic models. The fractional composition of biomass constituents in a human SNpc dopaminergic neuron is identical to that of the iDopaNeuro models (O’Brien and Sampson, 1965; Landolt et al., 1966; Norton et al., 1975; Banay-Schwartz et al., 1992; Preciat et al., 2021a) and comprises 39.6% lipid gDW, 56% protein gDW, 0.33% RNA gDW, 0.18% DNA gDW, and 3.96% other molecules gDW. We primarily focused on the turnover of amino acids and lipids, which together constitute over 90% of cellular constituents. Detailed calculations of these turnover constraints are provided in Supplementary File 1. These constraints were then used to restrict the main degradation pathways of the biomass precursors, representing their minimal consumption. Coupled reactions, which account for multiple degradation pathways from a single biomass precursor, were set to match those in the iDopaNeuro models for both synaptic and non-synaptic models.

To maintain mass balance, several specific exchange reactions were designated as active to simulate metabolite exchange between a neuron system and its environment. The essential amino acids for dopaminergic neurons, including histidine, isoleucine, leucine, lysine, methionine, phenylalanine, threonine, tryptophan, and valine, were contrainted for uptake only. Due to a dearth of experimentally measured metabolite uptake and secretion rates for *in vivo* dopaminergic neurons, exometabolomic data from *in vitro* dopaminergic neurons differentiated from human induced pluripotent stem cells (Preciat et al., 2021a) were used to constrain uptake and excretion of selected metabolites in all models. The uptake and secretion of additional metabolites were constrained using further evidence from *in vivo* neurobiochemical studies. For example, unconjugated bile acids such as cholic acid (CA), chenodeoxycholic acid (CDCA), and deoxycholic acid (DCA) have been detected in the rat brain (Higashi et al., 2017); reduced glutathione can convert to cysteinyl-glycine in astrocytes, which then supplies cysteine to neurons (Cakir et al., 2007); and lactate and ketones, including acetoacetate and -hydroxybutyrate, can be taken up by neurons and used as energy supplements (Hyder et al., 2006; Cunnane et al., 2020; Jensen et al., 2020; Omori et al., 2022; Yamagata, 2022). All activated exchange reactions capable of metabolite uptake were used as artificial media data and saved in the “mediaData” sheet for each model, as detailed in Supplementary Files 1–4.

**Specific constraints to differentiate the synaptic and non-synaptic components**. The constraints for the internal reactions were derived from experimental literature, particularly regarding to the enzyme activities in synaptic and non-synaptic mitochondria. For instance, the minimum and maximum enzyme activities measured in isolated synaptic and non-synaptic mitochondria were used to constrain the corresponding mitochondrial reactions, especially those involved in oxidative phosphorylation (Leong et al., 1984; Almeida et al., 1995; Davey and Clark, 1996; Davey et al., 1998). Additionally, the cerebral metabolic rates of glucose and oxygen in the putamen and substantia nigra were employed as maximum uptake values in the synaptic and non-synaptic models, respectively (Powers et al., 2008). Furthermore, enzyme activities related to glycolytic metabolism (Lowry and Passonneau, 1964), purine and pyrimidine metabolism (Maiuolo et al., 2016), amino acid metabolism (Vorstman et al., 2009), and lipid metabolism, including the synthesis of phosphatidylethanolamine (PE) and phosphatidylcholine (PC) (Roberti et al., 1980), were obtained from published studies and used to constrain the synaptic and non-synaptic models.

**Specific constraints to differentiate normal and PD status**. Evidence indicates that enzyme activities differ in patients with PD compared with healthy individuals, particularly in the mitochondria and those involved in dopamine metabolism (Bourne, 1968; Schapira et al., 1990; Stauch et al., 2014). For instance, Complex I activity has been reported to be reduced by 18-35% in post-mortem substantia nigra tissue from PD patients relative to control subjects (Schapira et al., 1990). Therefore, we set the maximum Complex I activity in PD models to 82% of that observed in control models. A well-known pathological characteristic of PD is the progressive loss of dopaminergic neurons and a decrease in dopamine levels in the SNpc (Bloem et al., 2021). It has been reported that the striatal dopamine concentration in individuals with PD is approximately one-quarter of that in control subjects (Bourne, 1968). Consequently, in this study, we assumed that dopamine release in the synaptic PD model is one-quarter of the release observed in the control model.

Moreover, to better differentiate PD and control models, we obtained data on maximum glucose and oxygen consumption in early-stage PD patients and age-matched controls who were studied while awake (Powers et al., 2008). The minimal glucose uptake was set at 25% of the maximum uptake in control models, reflecting the allocation of approximately 25% of glucose uptake to neurons (Hyder et al., 2006). For PD models, the minimum uptake of glucose and oxygen was set to zero due to the uncertainty surrounding minimal consumption in PD patients. Finally, the corresponding biochemical reactions of lysosomal -glucocerebrosidase were constrained based on the maximum GBA enzyme activities observed in induced pluripotent stem cell-derived (iPSC) neurons from both PD and control groups (Schöndorf et al., 2014).

It has been reported that 1 g of wet weight (WW) tissue corresponds to 4-6 mg of non-synaptic mitochondrial protein and 1-1.5 mg of synaptic mitochondrial protein in the rat striatum (Leong et al., 1984). In this study, we assumed that 1 g of WW gray matter corresponds to approximately 5 mg of non-synaptic mitochondrial protein and 1.25 mg of synaptic mitochondrial protein. When enzyme activities were reported in a mixed mitochondrial protein homogenate, 5 mg of mitochondrial protein per gram of WW gray matter was applied as an average. In cases where enzyme activity was measured in a whole protein homogenate, an average protein fraction of 9.9% of wet weight tissue was applied for unit conversion (O’Brien and Sampson, 1965). Due to the variability in reported enzyme activities, attributable to differences in species and experimental techniques, the higher reported value was chosen as the upper bound constraint to avoid overly constraining the model.

Furthermore, the residual energy consumption rate of human gray matter is estimated at 10 mol ATP/g/min (Attwell and Laughlin, 2001), which corresponds to approximately 106.2 mol/gDW/h. It has been reported that each human SNpc dopaminergic neuron generates about ten times as many synapses and has an axonal length roughly ten times greater than that of a rat neuron (Bolam and Pissadaki, 2012; Pissadaki and Bolam, 2013). Since our models employ rat synaptic and non-synaptic mitochondrial data to constrain most mitochondrial reactions, we reduced the minimum energy demand by a factor of ten. Specifically, the lower bound for ATP maintenance reaction (ATPM) was set at 10.62 mol/gDW/h, while the upper bound was established at 600 mol/gDW/h across all models. This upper bound corresponds to 10 Hz tonic firing activity and the 9.36 10^0^ ATP molecules per Hz of action propagation by 382,000 dopaminergic neurons within a volume of 6,280 mm in the dorsal striatum (Liss et al., 2001; Pissadaki and Bolam, 2013).

#### 2.1.2 Transcriptomic data

The ScRNA-seq data for human dopamine neurons (DA) were obtained from CELLxGENE^2^, a resource offering a suite of tools for exploring published single-cell data. We identified ScRNA-seq profiles for 22,048 DA neuron profiles from patients with PD and matched controls, which revealed 10 DA subgroups (Kamath et al., 2022). One of the DA subgroups, SOX6-AGTR1, is characterized by the expression of the genes SOX6 and AGTR1. This subgroup is primarily located in the ventral tier of the SNpc and is particularly vulnerable to degeneration in PD. Therefore, we used the data from this subgroup as our transcriptomic data to more effectively differentiate control and PD states in the synaptic and non-synaptic models of dopaminergic neurons.

Before being used as input data for the XomicsToModel pipeline, the transcriptomic data were preprocessed as follows. Initially, genes with a total count of zero were removed from both the PD and control matrices. The unique molecular identifier (UMI) count data were normalized using the median of ratios method (Anders and Huber, 2010; Chen et al., 2018), which involved dividing counts by sample-specific size factors determined from the median ratio of gene counts relative to the geometric mean of each gene. Finally, the mean expression value for each gene was calculated and used as gene weights in the XomicsToModel pipeline. All gene IDs were converted to Entrez IDs to better match those in the Recon3D Model. The preprocessed transcriptomic data for the control and PD groups is provided in Supplementary File 5. Threshold values for PD and control data were determined based on housekeeping genes (Hounkpe et al., 2021) to identify the most active genes, and these thresholds were subsequently used as input parameters for the XomicsToModel pipeline. Specifically, the threshold for both PD and control datasets was set to –3, as shown in Supplementary Figure 1.

#### 2.1.3 Modeling parameters

In this study, we used the “thermoKernel” algorithm within the XomicsToModel pipeline to extract thermodynamically flux-consistent models (Preciat et al., 2021b). The solver Gurobi v9.1.2 (Gurobi Optimization, LLC) was selected to solve linear optimisation problems. Additionally, the “fastcc” algorithm (Vlassis et al., 2014) was employed to check flux consistency. The specific parameters used to generate our models were set as follows: the parameter epsilon, which is used as a threshold to define a non-zero flux in the fastcc implementation, was set to 1e-4 (epsilon = 1e-4). The parameter weightsFromOmics, which uses preprocessed gene expression from transcriptomic data as a weight to incentivise or penalize the corresponding reactions, was set to true (weightsFromOmics = true). For the synaptic and non-synaptic components, manually assigned active and inactive genes and reactions were retained in case of conflicts between bibliomics and transcriptomic data. Consequently, the parameter curationOverOmics, which is used to resolve any conflicts between bibliomics and transcriptomic data, was also set to true (curationOverOmics = true) to prioritize bibliomics curation. The parameter activeOverInactive, which is used to resolve conflicts between active and inactive genes, was set to true (activeOverInactive = true) to prioritize active genes. The parameter transcriptomicThreshold, used to identify the most active genes in the transcriptomic data, was set to –3 (transcriptomicThreshold = –3). Other parameters were set to their default values, as described elsewhere (Preciat et al., 2021b).

### 2.2 Model analysis

#### 2.2.1 Entropic flux balance analysis

Flux balance analysis (FBA) is a widely used computational method for predicting the steady-state behavior of metabolic networks by calculating metabolic fluxes under defined constraints (Orth et al., 2010). However, FBA may generate thermodynamically infeasible fluxes within stoichiometrically balanced cycles that violate energy conservation and the second law of thermodynamics (Fleming et al., 2012). These infeasible fluxes can lead to unbounded metabolic fluxes in such cycles, even though mass balance and directionality constraints are satisfied. In this study, entropic flux balance analysis [EntropicFBA, (Fleming et al., 2012; Preciat et al., 2021a)] was used with default settings to compute an optimal, thermodynamically feasible solution for a biological objective function. This method enables the computation of optimal fluxes that satisfy steady-state mass conservation, energy conservation, and the second law of thermodynamics in genome-scale biochemical networks. EntropicFBA is represented by the following mathematical optimisation problem


(1) $$\begin{array}{cc} {\text{min}}_{v_{f},v_{r},w} & \text{g○}{\text{v}}_{f}^{T}\cdot \text{l}\,o\,g\,\left({\text{v}}_{f}\right)+\text{g○}{\text{v}}_{r}^{T}\cdot \text{l}\,o\,g\,\left({\text{v}}_{r}\right)+{\text{c}}_{e}^{T}\cdot \text{w}+\frac{1}{2}\,{\left(\text{v}-\text{h}\right)}^{T}\cdot \text{H}\cdot \left(\text{v}-\text{h}\right)\\ s.t. & \text{N}\cdot \left({\text{v}}_{f}-{\text{v}}_{r}\right)+\text{B}\cdot \text{w}=\text{b}:{\text{y}}_{N}\\ & \text{C}\cdot \left({\text{v}}_{f}-{\text{v}}_{r}\right)\leq \text{d}:{\text{y}}_{C}\\ & \text{l}\leq \left[{\text{v}}_{f}-{\text{v}}_{r};;\text{w}\right]\leq \text{u}:{\text{z}}_{v}\\ & 0\leq {\text{v}}_{f}:{\text{z}}_{v_{f}}\\ & 0\leq {\text{v}}_{r}:{\text{z}}_{v_{r}} \end{array}$$

where the parameter *g* is a strictly positive weight for internal flux entropy maximization (the default value is 2), ° denotes the entrywise (Hadamard) product of two vectors and ⋅ denotes the scalar product of two vectors. Since all the reversible reactions can be split into the forward and reverse directions, the net internal flux *v* can be expressed as the difference between the forward flux *v_f_* and the reverse flux *v_r_*, where *v* = *v*_*f*_ − *v*_*r*_. The terms $g^{○}\,v_{f}^{T}\cdot \text{log}\,\left(v_{f}\right)$ and $g^{○}\,v_{r}^{T}\cdot \text{log}\,\left(v_{r}\right)$ represent the entropy of unidirectional forward and reverse fluxes, respectively. External reactions are optimized with $c_{e}^{T}\cdot w$, where *c_e_* is a real-valued linear objective coefficient for external flux, and *w* is the external flux vector. The term $\frac{1}{2}\,{\left(v-h\right)}^{T}\cdot \text{H}\cdot \left(v-h\right)$ represents the quadratic penalty terms for the predicted fluxes from experimental or target flux data, where h is a vector representing experimental or target flux data, which serves as a reference for penalizing deviations from the data, and H is matrice representing penalties for predicted fluxes. The equality constraint *N* ⋅ (*v*_*f*_ − *v*_*r*_) + *B* ⋅ *w* = *b* represents mass balance, while the inequality constraints *C* ⋅ (*v*_*f*_ − *v*_*r*_) < *d* represents coupling between reaction fluxes. Box constraints implement lower and upper bounds on net fluxes, *l* ∈ ℝ^*n*^ and *u* ∈ ℝ^*n*^ respectively, or strictly non-negative constraints on internal unidirectional fluxes. This is a strictly convex optimisation problem that predicts unique optimal unidirectional forward and reverse fluxes. The dual variables (also called Lagrange multipliers, shadow prices, or dual prices) associated with each of these constraints, that is *y*_*N*_, *y*_*C*_, *z*_*v*_, *z*_*v*_*f*__, *z*_*v*_*r*__ respectively, represent how much the optimal value of the objective function would change if a constraint were slightly relaxed or tightened (Boyd and Vandenberghe, 2023). In particular the dual variable to the mass balance constraints, that is *y*_*N*_, is analogous to the chemical potential of each molecular species (Fleming et al., 2012). The Mosek v10.0.40 optimisation solver (MOSEK ApS) was employed to solve the nonlinear convex optimisation problems arising in EntropicFBA with the default parameters.

All the codes used in this study rely on the COBRA Toolbox 3.0 (Heirendt et al., 2019), including the “XomicsToModel” pipeline for model generation, the thermoKernel model extraction algorithm, and EntropicFBA algorithm for further analysis. The solvers, such as Gurobi v9.1.2 and Mosek v10.0.40, are all interfaced with the COBRA Toolbox 3.0.

#### 2.2.2 Model characteristics

After generating the models, we first performed validity checks on each model to evaluate basic metabolic functions (Heirendt et al., 2019). These checks involved verifying whether the model could generate ATP using different carbon sources and determining which basic human functions were feasible. Subsequently, we compared the metabolites, reactions, and genes in our models with those in the iDopaNeuro models (iDopaNeuroC and iDopaNeuroCT) to assess their consistency with general dopaminergic neuronal models.

#### 2.2.3 Bioenergetic analysis

To elucidate the bioenergetics differences between the synaptic and non-synaptic models under control and PD conditions, we calculated the flux distribution for each model using EntropicFBA, with the ATP maintenance (ATPM) as the objective function. Subsequently, we identified the biochemical reactions responsible for ATP contribution and consumption in each model. ATP contribution reactions were defined as those with net ATP generation with non-zero fluxes, while ATP consumption reactions exhibited net ATP consumption. The ATP contribution reactions were then classified into their respective subsystems to explore energy contribution differences among the models. To investigate energy contributions under varying energy demands, the ATPM constraint was set to values between 10 and 600 mol/gDW/h, reflecting a range from minimum to maximum energy demand. Comparisons among the models, particularly between the synaptic and non-synaptic models, and between the control and PD models, were performed to explore their bioenergetic differences.

Since the ongoing debate regarding oxygen and glucose consumption rates in the nigrostriatal region of PD patients compared to healthy controls (Powers et al., 2008; Borghammer et al., 2012), we conducted a sensitivity analysis of these models to assess the sensitivity of oxidative phosphorylation to variations in oxygen and glucose consumption. For each model, the flux of ATP synthesis via oxidative phosphorylation was calculated by varying the uptake constraints for oxygen or glucose from their lower bound to zero. Then, we compared how these changes in oxygen or glucose consumption affected ATP synthesis through oxidative phosphorylation between the control and PD models.

#### 2.2.4 Complex I inhibition analysis

Given that significantly reduced Complex I activity has been observed in PD patients, we conducted a Complex I inhibition analysis in the models to explore the differential sensitivity to Complex I inhibition between the synaptic and non-synaptic components. To simulate the inhibition of Complex I activity, we reduced the lower bound of Complex I reaction from 0% (no inhibition) to 100% (full inhibition) in each model and computed the resulting flux changes in ATP synthesis via oxidative phosphorylation under varying energy demands. Next, the flux changes between PD and control models were compared for both synaptic and non-synaptic components to assess their relative sensitivity to Complex I inhibition.

#### 2.2.5 Exploration of metabolite exchanges

Considering that selective vulnerability may be linked to dynamic metabolomic patterns in PD, we further investigated metabolite exchanges in PD models, thereby identifying either consistent or inconsistent metabolic changes with those observed in PD patients. Metabolites reproducibly altered in the cerebrospinal fluid (CSF) of PD patients, as reported in multiple independent metabolomic studies, were used to evaluate differences in the metabolite exchanges predicted by PD models (Luo et al., 2024). These metabolites, referred to as PD CSF metabolites in this study, were hypothesized to reflect metabolic dysfunctions in dopaminergic neurons. Therefore, PD CSF metabolites may serve as a basis for comparing the metabolic concentration changes observed in PD patients with the flux changes predicted by the PD models. Specifically, if a metabolite is increased in PD CSF, it may indicate lower uptake or higher excretion in the corresponding exchange reaction within the PD models. Conversely, a decrease in a metabolite in PD CSF may suggest higher uptake or lower excretion in the models. Subsequently, we explored the relative flux changes of exchange reactions for PD CSF metabolites in the synaptic and non-synaptic PD models to explore their consistency and inconsistency with the changes observed in PD CSF.

#### 2.2.6 Bioenergetic rescue analysis

Moreover, bioenergetic rescue analyses was performed on the PD models to explore potential therapeutic targets for dysfunctional dopaminergic neurons. These analyses aimed to identify reactions that could computationally restore the PD energy state and the dysfunctional metabolite exchanges to a control state. To identify reactions that significantly impact energy contribution, we employed the single-reaction flux inhibition method and single-reaction flux increaseing method.

In the single-reaction flux inhibition method, each reaction in the model was individually inhibited by setting its bound to zero, and the resulting ATP contribution was then recalculated to assess bioenergetic improvement. Reactions that restored glycolytic pathway and ATP synthase fluxes to levels approaching or exceeding those observed in the control under varying energy demands were identified as potential energy rescue reactions. Moreover, we assessed improvements in the fluxes of exchange reactions for PD CSF metabolites in the PD models to determine whether these rescue reactions could reverse or improve the flux alterations observed in the PD models relative to the corresponding control models. The most effective PD rescue reaction, especially those that could improve metabolite exchanges associated with dopamine release, was identified as potential therapeutic targets for PD patients. To better understand the impact of inhibited reactions, we also examined the changes in the chemical potential of the associated metabolites. In thermodynamics and biochemical systems, chemical potential represents the change in free energy that occurs when a small quantity of a substance is added or removed, where a negative value indicates a tendency to participate in reactions that release free energy, whereas a positive value implies a tendency to consume energy. Within the framework of EntropicFBA, the dual variable *y_N_* in the Equation 1, associated with the steady-state constraint, reflects the marginal cost of each metabolite required to maintain the steady-state condition. Therefore, the variable *y_N_* can be interpreted as representing the chemical potential of each metabolite, thereby providing insight into altered pathways after the reaction inhibition in the network. To better identify the reactions whose enhancement might improve bioenergetic performance and metabolite exchanges, we performed a single-reaction flux increasing analysis. In this method, the flux of each internal reaction in the PD models was individually increased by setting its bound to 1.1 times the original entropicFBA flux value (without any increasing). To predict perturbed fluxes resulting from increased reaction flux, we used flux entropy maximization as the objective for internal reactions. Simultaneously, the exchange reaction bounds derived from original entropicFBA flux (target fluxes) were replaced with the more relaxed generic bounds from the same reactions in the model to maintain similar PD molecular exchanges, and a quadratic penalty term was used to the objective to penalize deviation from the target fluxes (Preciat et al., 2021a). Similarly, bioenergetic improvements and changes in metabolite exchanges were assessed following the increase in the flux of each internal reaction.

## 3 Results

### 3.1 Model characteristics

**FIGURE 1:**
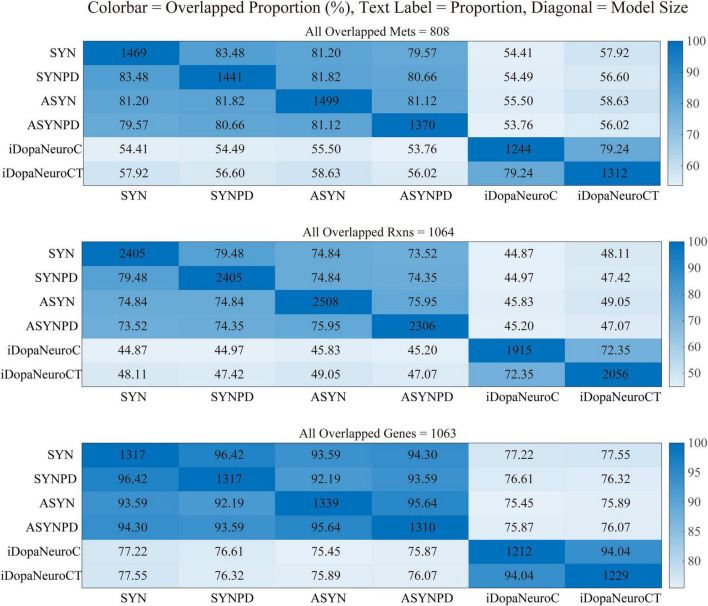
The overlapping metabolites, reactions, and genes shared between synaptic and non-synaptic models.

Four thermodynamically flux-consistent models were generated: a synaptic control model (SYN), a synaptic PD model (SYNPD), a non-synaptic control model (ASYN), and a non-synaptic PD model (ASYNPD). These models successfully passed validity checks based on approximately 80 basic metabolic functions, such as generating ATP from several carbon sources and performing basic lipid and amino acid metabolism, as detailed in Supplementary File 6. The general model statistics, such as the total number of reactions, metabolites, genes, and other characteristics, are summarized in Table 1. The four models are of similar size, each containing approximately 750 unique metabolites, 2,400 reactions, and 1,300 metabolic genes, which span over 70 pathways and 9 subcellular compartments. The overlap of metabolites, reactions, and genes between the synaptic and non-synaptic models, as well as the iDopaNeuro models, is illustrated in Figure 1. More than 70% of the metabolites and reactions, as well as 90% of the genes from the iDopaNeuro models, are present in both the synaptic and non-synaptic models.

**TABLE 1:** General model statistics for synaptic and non-synaptic models.

	SYN	SYNPD	ASYN	ASYNPD
Total number of genes	1,317	1,317	1,339	1,310
Total number of reactions	2,405	2,405	2,508	2,306
Total number of metabolites	761	753	767	728
Total number of transport reactions	1,040	1,028	1,074	981
Total number of exchange reactions	227	237	231	212
Total number of subsystems	76	75	78	75
Total number of compartments	9	9	9	9
Total number of Genes in the mitochondria	377	391	414	384
Total number of reactions in the mitochondria	519	532	555	508
Total number of metabolites in the mitochondria	296	303	306	281

### 3.2 Bioenergetic differences between synaptic and non-synaptic models

**FIGURE 2:**
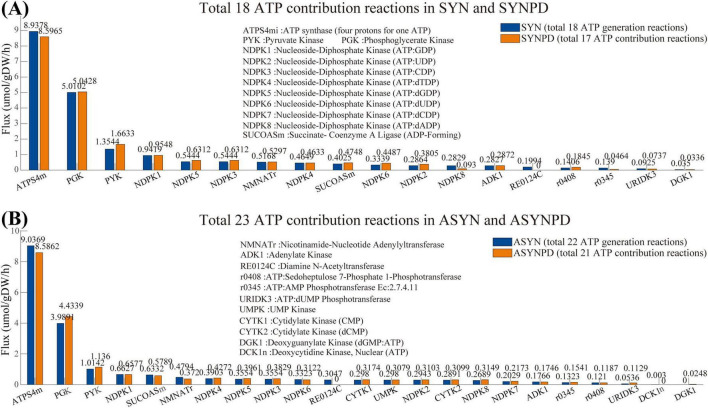
Flux comparison of reactions contributing to ATP production in synaptic and non-synaptic models under control and PD conditions. **(A)** Comparison of ATP contribution reactions in control and PD synaptic models, with the horizontal axis representing ATP contribution reactions and the vertical axis showing the corresponding flux values. **(B)** Comparison of ATP contribution reactions in control and PD non-synaptic models. In all models, ATP is primarily produced via oxidative phosphorylation (ATPS4mi) and glycolysis, particularly through phosphoglycerate kinase (PGK) and pyruvate kinase (PYK).

Figure 2 presents a comparison of ATP contribution reactions under control and PD conditions for both synaptic and non-synaptic models at a minimum energy demand of 10.62 mol/gDW/h. In both model types, ATP is primarily generated by ATP synthase via oxidative phosphorylation (ATPS4mi) and by glycolysis, particularly through phosphoglycerate kinase (PGK) and pyruvate kinase (PYK). Additional ATP generating pathways include the citric acid cycle (SUCOASm), nucleotide interconversion (e.g., NDPK1, ADK1, and CYTK1), NAD metabolism (NMNATr), and the pentose phosphate pathway (r0408 and RE0124C).

**FIGURE 3:**
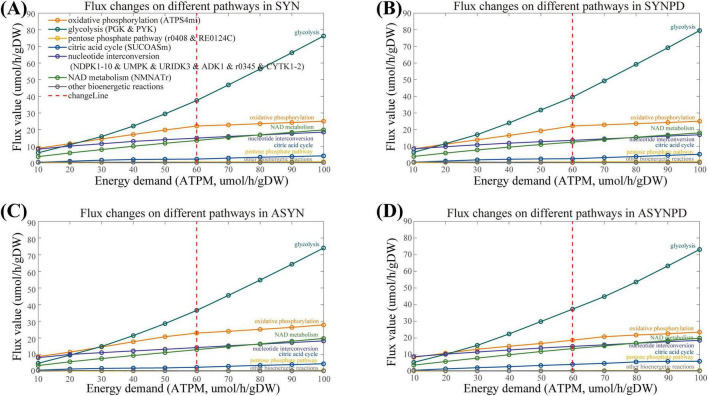
Flux changes across various subsystems in both synaptic and non-synaptic models, with energy demand (ATPM) ranging from 10 to 100 μmol/gDW/h. **(A)** Flux changes across different subsystems in the synaptic control model under varying energy demands. **(B)** Flux changes across different subsystems in the synaptic PD model under varying energy demands. **(C)** Flux changes across different subsystems in the non-synaptic control model under varying energy demands. **(D)** Flux changes across different subsystems in the non-synaptic PD model under varying energy demands. The flux from oxidative phosphorylation approaches a plateau at an energy demand of 60 μmol/gDW/h, with only slight increases beyond this threshold, whereas glycolytic flux continues to rise as energy demand increases.

The comparison of ATP contribution between the synaptic and non-synaptic models with energy demands (ATPM) ranging from minimum to maximum (10–600 mol/gDW/h), revealed a noticeable shift from oxidative phosphorylation to glycolysis (Supplementary Figure 2). This shift can be attributed to specific mitochondrial constraints imposed by available experimental data, which limit the capacity of mitochondria to generate additional energy via oxidative phosphorylation. Notably, the flux from oxidative phosphorylation reaches a near-plateau at an ATPM of 60 mol/gDW/h and increases only slightly beyond this point (see Figure 3). Therefore, we then mainly explored the energy contributions within the ATPM range of 100 mol/gDW/h.

**FIGURE 4:**
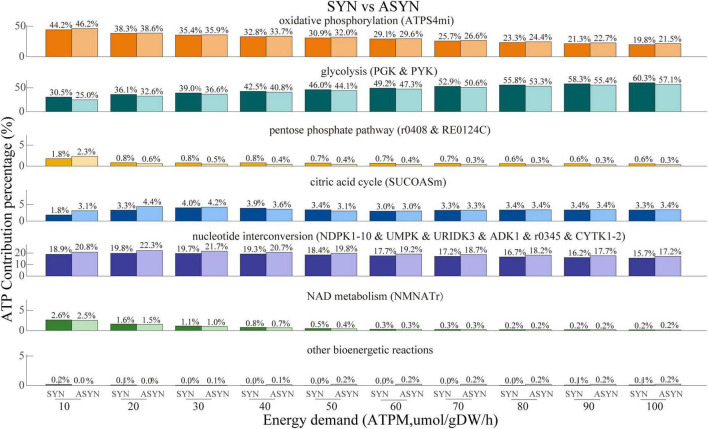
ATP contribution proportions across various subsystems with energy demand ranging from 10 to 100 μmol/gDW/h in both synaptic and non-synaptic models. Compared to the non-synaptic model, the synaptic model exhibited a lower energy contribution from oxidative phosphorylation, citric acid cycle, and nucleotide interconversion, but a higher energy contribution primarily from glycolysis.

**FIGURE 5:**
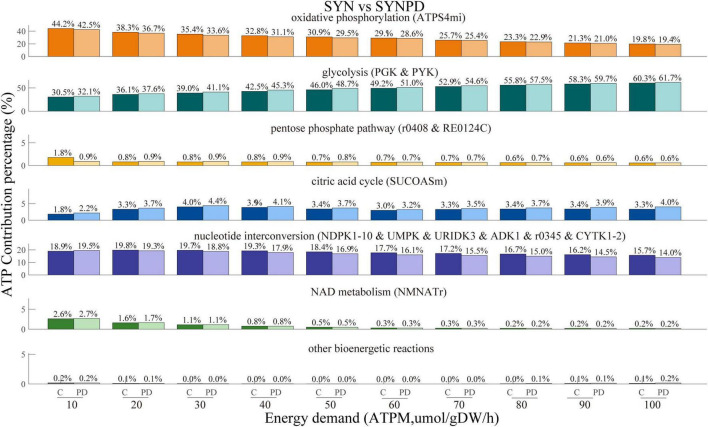
ATP contribution proportions across different subsystems, with energy demand ranging from 10 to 100 μmol/gDW/h, in synaptic control and PD models. In the synaptic PD model, oxidative phosphorylation contributes less to ATP production than in the control model, while glycolysis and the citric acid cycle contribute more.

The comparison of ATP contribution between the synaptic and non-synaptic control models under the ATPM range of 100 mol/gDW/h is shown in Figure 4. The synaptic model exhibited relatively lower ATP contributions from oxidative phosphorylation, the pentose phosphate pathway, the citric acid cycle, and nucleotide interconversion compared to the non-synaptic model. In contrast, glycolysis and NAD metabolism contributed more, particularly under relatively low energy demands. Compared with the control model, the synaptic PD model demonstrated a lower contribution of oxidative phosphorylation to ATP generation, while glycolysis and the citric acid cycle contributed more, as shown in Figure 5. Similarly, the non-synaptic PD model displayed significantly decreased energy contributions from oxidative phosphorylation and increased contributions from glycolysis and the citric acid cycle relative to the control model (Supplementary Figure 3).

The results of the sensitivity analysis for oxygen and glucose are shown in Supplementary Figure 4. Both synaptic and non-synaptic PD models showed reduced energy contributions from oxidative phosphorylation compared to their corresponding control models, irrespective of oxygen uptake values. When oxygen uptake was reduced from its highest values, the synaptic PD model exhibited an earlier decline in ATP synthesis flux than the non-synaptic PD model. Additionally, glucose metabolism maintained stable ATP synthesis via oxidative phosphorylation across all models, as glucose was substituted with alternative carbon sources, particularly lactate and galactose (Supplementary Figure 5).

### 3.3 Complex I inhibition analysis

**FIGURE 6:**
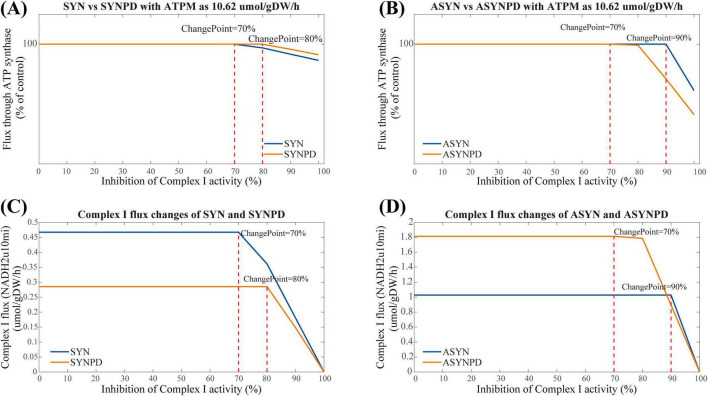
Complex I inhibition in synaptic and non-synaptic models at a minimum ATPM of 10.62 μmol/gDW/h. **(A)** Comparison of Complex I inhibition between control and PD synaptic models. **(B)** Comparison of Complex I inhibition between control and PD non-synaptic models. **(C)** Flux changes of Complex I under varying inhibition in the synaptic models. **(D)** Flux changes of Complex I under varying inhibition in the non-synaptic models. ATP synthesis via oxidative phosphorylation remained stable up to 70% Complex I inhibition in the synaptic control model and 80% in the synaptic PD model, reflecting higher sensitivity in the PD condition. In the non-synaptic models, ATP synthesis remained stable up to 90% inhibition in the control, with reductions observed at 70% in the PD model.

The results of Complex I inhibition in the control and PD models at a minimum energy demand are shown in Figure 6. In the synaptic control model, ATP synthesis via oxidative phosphorylation remained stable until 70% inhibition of Complex I activity, whereas in the synaptic PD model, a decline in ATP synthesis via oxidative phosphorylation was observed at 80% inhibition (Figure 6A). The non-synaptic models exhibited a longer plateau phase than the synaptic models. Specifically, in the non-synaptic control model, ATP synthesis via oxidative phosphorylation began to decline at 90% inhibition of Complex I activity, whereas in the non-synaptic PD model, a reduction in ATP synthesis was evident at 70% inhibition, with significant changes apparent at 80% inhibition (Figure 6B). Detailed flux changes for Complex I under different levels of inhibition in each model are shown in Figures 6C,D).

**FIGURE 7:**
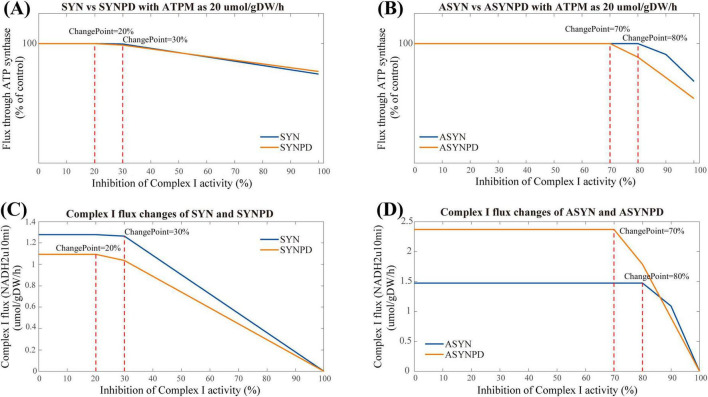
Complex I inhibition in synaptic and non-synaptic models at an ATPM of 20 μmol/gDW/h. **(A)** Comparison of Complex I inhibition between control and PD synaptic models. **(B)** Comparison of Complex I inhibition between control and PD non-synaptic models. **(C)** Flux changes of Complex I under varying inhibition levels in synaptic models. **(D)** Flux changes of Complex I under varying inhibition levels in non-synaptic models. In the synaptic PD model, an earlier energy inflection point is observed, with lower Complex I flux than in the control model. Changes in ATP synthesis occur at 30% Complex I inhibition in the control model and at 20% in the PD model. In the non-synaptic models, ATP synthesis alterations are evident at 70% inhibition in the control model and 80% in the PD model.

Nevertheless, the observation in the synaptic models differ from the corresponding experimental findings (Davey et al., 1998), where ATP synthesis through oxidative phosphorylation decreased linearly once Complex I activity was inhibited by more than 25% of its control activity in isolated synaptic mitochondria. Consequently, we performed the Complex I inhibition under varying energy demand for each model. The results of Complex I inhibition with an ATPM of 20 mol/gDW/h align more closely with experimental findings (Davey et al., 1998), as shown in Figure 7. An earlier decline in ATP synthesis via oxidative phosphorylation was observed in the synaptic models, with changes detected at 30% inhibition in the synaptic control model, while in the synaptic PD model, these changes occurred at a lower threshold of 20% inhibition. In the non-synaptic models, changes in ATP synthesis were observed at 70 and 80% inhibition of Complex I for the control and PD models, respectively.

This difference may partly result from the predefined constraints on mitochondrial reactions imposed by available experimental data, particularly on Complex I. In the synaptic models, Complex I reaches maximum activity at an ATPM value of 30 mol/gDW/h, while the non-synaptic models do not exhibit a significant plateau in Complex I activity due to relatively relaxed constraints (Supplementary Figure 6). Therefore, varying energy demands have a significant impact on the observed performance of Complex I inhibition in the synaptic models.

### 3.4 Comparison of metabolite exchanges

A total of 36 metabolites were significantly changed in the CSF of PD patients across eight metabolomic studies from a previous meta-analysis (Luo et al., 2024; Supplementary File 7). Seventeen of these metabolites have corresponding exchange reactions that enable either uptake or excretion in both synaptic and non-synaptic models. In synaptic models, the defined constraints and predicted flux values for these exchange reactions are shown in Table 2.

**TABLE 2:** Flux changes of 17 exchange reactions between control and PD in synaptic models.

Reaction ID	Metabolite name	Changes in PD CSF	Bounds in SYN	Flux in SYN	Bounds in SYNPD	Flux in SYNPD	Changes in PD model	Consistency
EX_dopa[e]	Dopamine	Increased (Yilmaz et al., 2020)	[0.0007, 1,000]	1.0826	[0.0002, 1,000]	0.3840	Lower excrete	No
EX_glc_D[e]	D-Glucose	Increased (Trezzi et al., 2017); decreased (Lewitt et al., 2013)	[–3.01, –0.7525]	–0.7525	[–3.69, 0]	–0.3146	Lower uptake	Yes
EX_trp_L[e]	L-Tryptophan	Decreased (Trupp et al., 2014)	[–0.8357, 0]	–0.8357	[–0.8357, 0]	–0.8357	Same	–
EX_met_L[e]	L-Methionine	Increased (Yilmaz et al., 2020)	[–1.8909, 0]	–1.8909	[–1.8909, 0]	–1.2779	Lower uptake	Yes
EX_asn_L[e]	L-Asparagine	Increased (Yilmaz et al., 2020)	[0.4867, 1000]	0.4867	[0.4867, 1000]	0.4867	Same	–
EX_orn[e]	Ornithine	Increased (Wuolikainen et al., 2016)	[25.3657, 1000]	25.3657	[25.3657, 1000]	25.3657	Same	–
EX_gln_L[e]	L-Glutamine	Increased (Willkommen et al., 2018; Yilmaz et al., 2020); decreased (Trupp et al., 2014)	[–60.5564, 0]	–16.6588	[-60.5564, 0]	–16.6238	Lower uptake	Yes
EX_lys_L[e]	L-Lysine	Increased (Wuolikainen et al., 2016)	[–100, 0]	–15.0386	[–100, 0]	–14.9391	Lower uptake	Yes
EX_his_L[e]	L-Histidine	Increased (Wuolikainen et al., 2016; Yilmaz et al., 2020)	[–27.05, 0]	–6.3213	[–27.05, 0]	–7.2753	Higher uptake	No
EX_ile_L[e]	L-Isoleucine	Increased (Wuolikainen et al., 2016)	[–19.49, 0]	–8.4975	[–19.49, 0]	–8.4975	Same	–
EX_Lpipecol[e]	L-Pipecolic acid	Decreased (Lewitt et al., 2013)	[0, 1000]	0	[0, 1000]	0	Same	–
EX_cit[e]	Citric acid	Increased (Yilmaz et al., 2020)	[–1000, 1000]	0.2540	[–1000, 1000]	0.2540	Same	–
EX_4mop[e]	Ketoleucine	Increased (Wuolikainen et al., 2016)	[–1000, 1000]	–0.1546	[–1000, 1000]	–0.1951	Higher uptake	No
EX_ptrc[e]	Putrescine	Increased (Yilmaz et al., 2020; Plewa et al., 2021)	[–1000, 1.6468]	–0.1268	[–1000, 1.6468]	–0.1268	Same	–
EX_glyc_R[e]	Glyceric acid	Decreased (Trezzi et al., 2017)	[0.2876, 1000]	0.2876	[0.2876, 1000]	0.2876	Same	–
EX_urea[e]	urea	Decreased (Trezzi et al., 2017)	[0, 1000]	0.2291	[0, 1000]	0.1597	Lower excrete	Yes
EX_lac_L[e]	L-Lactic acid	Increased (Yilmaz et al., 2020)	[-6.48, 0.83898]	–0.3649	[-6.48, 0.83898]	–0.2427	Lower uptake	Yes

Flux changes in six exchanged metabolites in the synaptic PD model are consistent with the metabolic alterations observed in the CSF of PD patients. The relatively lower uptake predicted in the synaptic PD model are consistent with the increased levels of methionine (Yilmaz et al., 2020), glutamine (Willkommen et al., 2018; Yilmaz et al., 2020), lysine (Wuolikainen et al., 2016), and lactate (Yilmaz et al., 2020) observed in the PD CSF, while the reduced excretion of urea corresponds to decreased urea levels (Trezzi et al., 2017). The predicted decrease in dopamine flux in the synaptic PD model supports our hypothesis of reduced dopamine release in the PD state.

Although glucose levels in PD CSF have been reported inconsistently across studies, the synaptic PD model predicted lower glucose uptake compared to the synaptic control model. However, higher uptake of ketoleucine and histidine predicted in the synaptic PD model contrasts with the increased levels reported in the PD CSF (Wuolikainen et al., 2016; Yilmaz et al., 2020). Additionally, although increased CSF levels of tryptophan (Trupp et al., 2014), asparagine (Yilmaz et al., 2020), ornithine (Wuolikainen et al., 2016), isoleucine (Wuolikainen et al., 2016), citric acid (Yilmaz et al., 2020), pipecolic acid (Lewitt et al., 2013), and putrescine (Yilmaz et al., 2020; Plewa et al., 2021), along with the decreased level of glyceric acid (Trezzi et al., 2017) have been reported in PD CSF, these changes were not reflected in the synaptic PD model.

The flux changes for these exchange reactions in the non-synaptic PD model are shown in Table 3. Notably, no flux is associated with dopamine release in the non-synaptic models, as the corresponding reactions have been manually inhibited. The predicted flux changes in the uptake of glutamine, lysine, histidine, lactate, and ketoleucine, as well as the excretion of glyceric acid in the non-synaptic PD model, are consistent with changes observed in PD CSF. However, the predicted changes in methionine, pipecolic acid, ketoleucine, and urea are inconsistent with the observations in PD CSF. Similarly, the fluxes of several metabolites, including tryptophan, asparagine, isoleucine, ornithine, putrescine, and citric acid, remain unchanged between the non-synaptic control and PD models.

**TABLE 3:** Flux changes of 17 exchange reactions between control and PD in non-synaptic models.

Reaction ID	Metabolite Name	Changes in PD CSF	Bounds in ASYN	Flux in ASYN	Bounds in ASYNPD	Flux in ASYNPD	Changes in PD model	Consistency
EX_dopa[e]	Dopamine	Increased (Yilmaz et al., 2020)	[0, 1,000]	0	[0, 1,000]	0	No excrete	–
EX_glc_D[e]	D-Glucose	Increased (Trezzi et al., 2017); decreased (Lewitt et al., 2013)	[–1.93, –0.4825]	–0.4825	[–2.24, 0]	–0.2792	Lower uptake	Yes
EX_trp_L[e]	L-Tryptophan	Decreased (Trupp et al., 2014)	[–0.8357, 0]	–0.8357	[–0.8357, 0]	–0.8357	Same	–
EX_met_L[e]	L-Methionine	Increased (Yilmaz et al., 2020)	[–1.8909, 0]	–1.1715	[–1.8909, 0]	–1.2528	Higher uptake	No
EX_asn_L[e]	L-Asparagine	Increased (Yilmaz et al., 2020)	[0.4867, 1,000]	0.4867	[0.4867, 1,000]	0.4867	Same	–
EX_orn[e]	Ornithine	Increased (Wuolikainen et al., 2016)	[25.3657, 1,000]	25.3657	[25.3657, 1,000]	25.3657	Same	–
EX_gln_L[e]	L-Glutamine	Increased (Willkommen et al., 2018; Yilmaz et al., 2020); decreased (Trupp et al., 2014)	[–60.5564, 0]	–17.0832	[–60.5564, 0]	–16.8687	Lower uptake	Yes
EX_lys_L[e]	L-Lysine	Increased (Wuolikainen et al., 2016)	[–100, 0]	–19.2610	[–100, 0]	–18.9466	Lower uptake	Yes
EX_his_L[e]	L-Histidine	Increased (Wuolikainen et al., 2016; Yilmaz et al., 2020)	[–27.05, 0]	–6.9666	[–27.05, 0]	–5.9576	Lower uptake	Yes
EX_ile_L[e]	L-Isoleucine	Increased (Wuolikainen et al., 2016)	[–19.49, 0]	–8.4975	[–19.49, 0]	–8.4975	Same	–
EX_Lpipecol[e]	L-Pipecolic acid	Decreased (Lewitt et al., 2013)	[0, 1,000]	0.8811	[0, 1,000]	1.0950	Higher excrete	No
EX_cit[e]	Citric acid	Increased (Yilmaz et al., 2020)	[–1000, 1000]	0.2540	[–1,000, 1,000]	0.2540	Same	–
EX_4mop[e]	Ketoleucine	Increased (Wuolikainen et al., 2016)	[–1,000, 1,000]	–0.5428	[–1,000, 1,000]	–0.5550	Higher uptake	No
EX_ptrc[e]	Putrescine	Increased (Yilmaz et al., 2020; Plewa et al., 2021)	[–1,000, 1.6468]	–0.1268	[–1,000, 1.6468]	–0.1268	Same	–
EX_glyc_R[e]	Glyceric acid	Decreased (Trezzi et al., 2017)	[0.2876, 1,000]	0.3131	[0.2876, 1,000]	0.2876	Lower excrete	Yes
EX_urea[e]	Urea	Decreased (Trezzi et al., 2017)	[0, 1,000]	0.0624	[0, 1,000]	0.0882	Higher excrete	No
EX_lac_L[e]	L-Lactic acid	Increased (Yilmaz et al., 2020)	[–6.48, 0.83898]	–1.1569	[–6.48, 0.83898]	–0.6983	Lower uptake	Yes

### 3.5 Bioenergetic rescue analysis

#### 3.5.1 Single-reaction flux inhibition

Bioenergetic rescue analyses were conducted on the PD models to identify reactions capable of computationally improving the PD energy state and metabolite exchanges, thereby exploring potential therapeutic targets for dysfunctional dopaminergic neurons. The inhibition of 34 reactions in the synaptic PD model and 23 reactions in the non-synaptic PD model were identified that could improve the corresponding PD energy state, particularly with respect to oxidative phosphorylation (Supplementary File 8). However, only one reaction, mitochondrial phenylalanine transaminase (PHETA1m), that is


(2) Oxoglutaricacid+L-Phenylalanine<=>

L-Glutamic acid+Phenylpyruvic⁢acid

was identified as having the potential to improve the bioenergetic performance and enhance metabolite exchanges in the synaptic PD model, as shown in Supplementary File 9. In contrast, none of the inhibited reactions in the non-synaptic PD model were able to enhance both the metabolite exchanges and energy production under varying energy demands.

The predicted changes in chemical potential (strictly, the dual variable to the mass balance constraints) for each metabolite involved in the PHETA1m reaction of rescued synaptic PD model (PHETA1m inhibition) provide insight into altered metabolite concentrations within the network, as shown in Supplementary File 10. In conjunction with the observed reaction flux changes, increased levels of oxoglutaric acid, phenylalanine, and glutamic acid underscore the activation of alternative amino acid metabolism in the rescued PD model, such as lysine and isoleucine metabolism, as supported by increased fluxes in related reactions (Supplementary File 9). These alternative amino acid metabolic pathways further enhanced oxidative phosphorylation through the electron transfer flavoprotein (ETF) coupling pathway, as evidenced by increased fluxes in the corresponding reactions, particularly those mediated by electron transfer flavoprotein-ubiquinone oxidoreductase.

### 3.5.2 Single-reaction flux increasing

The single-reaction flux increasing analysis better target the reactions that enhancing the PD energy state and metabolite exchanges. The analysis results showed only the internal reaction, mitochondrial ornithine transaminase reaction (ORNTArm), that is


(3) Oxoglutaricacid+Ornithine<=>

L-Glutamic acid+L-Glutamicgamma-semialdehyde

could improve the bioenergetic performance and metabolite exchanges for both synaptic and non-synaptic PD model, as shown in Supplementary File 11. Table 4 presents flux improvements in ATP synthesis and metabolite exchanges following an increase in ORNTArm in the synaptic PD model. An increased flux in ATP synthesis was observed in the rescued synaptic PD model (ORNTArm increased), highlighting the improved bioenergetic performance on oxidative phosphorylation. Additionally, the rescued synaptic PD model predicted improved exchange fluxes for glutamine, lysine, histidine, ketoleucine, urea, and lactate compared with the original synaptic PD model. However, no improvement has been observed in the exchange of dopamine and glutamine. Moreover, Supplementary Figure 7 compares energy contributions under varying energy demands between the synaptic control and rescued PD models. The results demonstrate a stable bioenergetic improvement in the rescued synaptic PD model, associated with increased ORNTArm, irrespective of energy demand. In conjunction with other reaction flux changes (Supplementary File 12), the increased flux through ORNTArm, which catalyses the conversion of glutamate-5-semialdehyde and glutamate to ornithine and α−ketoglutarate in the model, enhanced the activity of the urea cycle and lysine metabolism in the rescued synaptic PD model. This, in turn, further increased oxygen consumption, thereby enhancing the respiratory chain.

**TABLE 4:** Flux improvement of ATP synthase and exchange reactions in the rescued synaptic PD model with the increased flux of the mitochondrial ornithine transaminase reaction.

Reaction ID	Description	Changes in PD CSF	Flux in SYN	Flux in original SYNPD	Flux in rescued SYNPD	Improvement
ATPS4mi	ATP synthase	–	8.9379	8.5965	9.4468	Yes
EX_dopa[e]	Exchange of Dopamine	Increased (Yilmaz et al., 2020)	1.0826	0.3840	0.3118	No
EX_met_L[e]	Exchange of L-Methionine	Increased (Yilmaz et al., 2020)	–1.8909	–1.2779	–1.2324	No
EX_gln_L[e]	Exchange of L-Glutamine	Increased (Willkommen et al., 2018; Yilmaz et al., 2020); decreased (Trupp et al., 2014)	–16.6588	–16.6238	–17.5047	Yes
EX_lys_L[e]	Exchange of L-Lysine	Increased (Wuolikainen et al., 2016)	–15.0386	–14.9391	–15.1006	Yes
EX_his_L[e]	Exchange of L-Histidine	Increased (Wuolikainen et al., 2016; Yilmaz et al., 2020)	–6.3213	–7.2753	–6.9867	Yes
EX_4mop[e]	Exchange of Ketoleucine	Increased (Wuolikainen et al., 2016)	–0.1546	–0.1951	–0.1687	Yes
EX_urea[e]	Exchange of urea	Decreased (Trezzi et al., 2017)	0.2291	0.1597	0.3223	Yes
EX_lac_L[e]	Exchange of L-Lactic acid	Increased (Yilmaz et al., 2020)	–0.3649	–0.2427	–0.3285	Yes

Table 5 shows the flux improvement of ATP synthesis and metabolite exchanges in the non-synaptic PD model. The improved exchange fluxes for glutamine, lysine, histidine, and lactate were observed in the rescued non-synaptic PD model. Furthermore, the bioenergetic improvement in oxidative phosphorylation was predominantly observed under low energy demand in the rescued non-synaptic PD model, as indicated in Supplementary Figure 8. Similarly to the rescued synaptic PD model, the increase in ORNTArm was associated with alterations in the urea cycle and an increase in oxygen uptake in the non-synaptic PD model (Supplementary File 12).

**TABLE 5:** Flux improvement of ATP synthase and exchange reactions in the rescued non-synaptic PD model with the increased flux of the mitochondrial ornithine transaminase reaction.

Reaction ID	Metabolite name	Changes in PD CSF	Flux in ASYN	Flux in original ASYNPD	Flux in rescued ASYNPD	Improvement
ATPS4mi	ATP synthase	–	8.5862	9.48623	9.5930	Yes
EX_gln_L[e]	Exchange of L-Glutamine	Increased (Willkommen et al., 2018; Yilmaz et al., 2020); decreased (Trupp et al., 2014)	–17.0832	–16.8687	–17.4678	Yes
EX_lys_L[e]	Exchange of L-Lysine	Increased (Wuolikainen et al., 2016)	–19.2610	–18.9466	–19.7937	Yes
EX_his_L[e]	Exchange of L-Histidine	Increased (Wuolikainen et al., 2016; Yilmaz et al., 2020)	–6.9666	–5.9576	–6.1757	Yes
EX_glyc_R [e]	Exchange of Glyceric acid	Decreased (Trezzi et al., 2017)	0.3131	0.2876	0.2876	No
EX_lac_L[e]	Exchange of L-Lactic acid	Increased (Yilmaz et al., 2020)	–1.1569	–0.6983	–0.7279	Yes

## 4 Discussion

### 4.1 Bioenergetic differences between models

A total of four models were generated to represent the synaptic and non-synaptic components of dopaminergic neurons under control and PD conditions. Although these models contain similar metabolites, reactions, and genes, they differed in their bioenergetic performance and metabolite exchange profiles. Regarding bioenergetic performance, all models predicted that oxidative phosphorylation plays a significant role under relatively lower energy demand, while glycolysis predominates when energy demand exceeded mitochondrial constraints. This shift highlights that mitochondrial contributions are limited by the predefined constraints derived from the available experimental data for both synaptic and non-synaptic mitochondria. Although these constraints, primarily based on rat or mouse mitochondrial experiments, restrict the exploration of energy dynamics under high energy demands, the bioenergetic differences observed within these constraints between models provide valuable insights into their relatively distinct bioenergetic features.

The synaptic models predicted a relatively lower ATP contribution from oxidative phosphorylation and the citric acid cycle compared with the non-synaptic models regardless of energy demand, suggesting reduced mitochondrial activities. This finding appears to conflict with our hypothesis that the synaptic component consumes and generates more energy through oxidative phosphorylation for dopamine release. The lower mitochondrial activities in the synaptic models, compared to the non-synaptic models, are consistent with the enzyme activities measured in the mitochondria of the synaptosomal fraction compared to free mitochondria (non-synaptic component) (Villa et al., 1989; Davey and Clark, 1996; Davey et al., 1998), which were used to generate models. The lower enzyme activities in synaptosomal mitochondria relative to free mitochondria may be due to the higher buoyant density of non-synaptic mitochondria and the inherent heterogeneity of synaptic mitochondria (Lai and Clark, 1976). Additionally, the widespread distribution of synaptic terminals may result in a lower density of synaptic mitochondria within individual units, further contributing to the observed low enzyme activities in synaptosomal mitochondria.

The predicted lower ATP contribution from the pentose phosphate pathway (PPP) and nucleotide interconversion in the synaptic models may indicate reduced antioxidative capacity and decreased nucleotide-related biological processes compared with the non-synaptic models. The PPP is a metabolic pathway parallel to glycolysis that converts glucose-6-phosphate into pentoses, producing ribose-5-phosphate and NADPH, which plays a key role in anabolic biosynthesis and redox homeostasis (Tu et al., 2019). In neural cells, the PPP primarily supplies cytosolic NADPH, essential for the antioxidative defense of brain cells through glutathione (GSH) redox cycling (Lajtha and Sylvester, 2008). Nucleotide interconversion, the process of converting one type of nucleotide into another, ensures an adequate supply for critical functions, such as DNA and RNA synthesis, energy transfer, and signal transduction. The reduced energy contribution from nucleotide interconversion in the synaptic models is consistent with the reduced activity of the citric acid cycle (where GTP is converted into ATP to meet energy requirements). Therefore, the higher ATP contribution from glycolysis predicted in the synaptic models may serve as a compensatory mechanism for insufficient energy production.

Compared to the control models, both synaptic and non-synaptic PD models predicted a lower ATP contribution from oxidative phosphorylation regardless of energy demand. The reduced ATP contribution from oxidative phosphorylation in the PD models supports the hypothesis of impaired electron transport chain in the mitochondria of dopaminergic neurons in PD patients (Dias et al., 2013; Zanfardino et al., 2021). Additionally, the decreased energy contribution from the PPP predicted in PD models under lower energy demand is consistent the decreased levels of glucose-6-phosphate dehydrogenase and 6-phosphogluconate dehydrogenase, key enzymes of the PPP, observed in the putamen and cerebellum of PD patients (Dunn et al., 2014; Camandola and Mattson, 2017). However, under increased energy demands, no significant difference in PPP flux was observed between the control and PD models, likely due to its low contribution to overall ATP generation. Moreover, the higher energy contributions from glycolysis and the citric acid cycle predicted in both synaptic and non-synaptic PD models may suggest a compensatory mechanism for maintaining energy production in the PD state (Travaglio et al., 2023).

The analysis of Complex I inhibition further emphasizes the bioenergetic differences between these two neuronal components. Under the defined mitochondrial constraints, the response to Complex I inhibition in the synaptic models is strongly influenced by energy demand. When energy demand increases, the synaptic models showed greater sensitivity to Complex I inhibition than the non-synaptic models, particularly under PD condition, with an earlier energy inflection point. In contrast, the non-synaptic models maintain relatively stable performance under Complex I inhibition regardless of energy demand. These differences in the models may suggest that the Complex I activity of synaptic component is more sensitive and vulnerable in relatively high energy environments than the non-synaptic component. Moreover, it may also indicate that the isolated mitochondria or cultured neurons may exhibit distinct energy dynamics under varying energy demands, potentially contributing to the observed experimental heterogeneity.

During the oxygen sensitivity analysis, the synaptic models predicted higher ATP synthesis via oxidative phosphorylation than the non-synaptic models when oxygen uptake was maintained above the predicted oxygen uptake, indicating a greater sensitivity of oxygen uptake in the synaptic models. This result also indicates a predefined minimum oxygen uptake may significantly influence the energy performance in the synaptic models. Furthermore, a comparison within the synaptic models reveals that the synaptic PD model exhibits higher sensitivity, with its energy inflection point occurring earlier than that of the control model as oxygen uptake declines. In contrast, the glucose sensitivity analysis indicated stable ATP synthesis via oxidative phosphorylation, suggesting that under certain conditions, other substrates can serve as the principal carbon source (Camandola and Mattson, 2017).

### 4.2 Differences of metabolite exchanges

The consistent and inconsistent metabolite exchanges observed in the synaptic and non-synaptic PD models may reflect distinct metabolic patterns between these neuronal components. In both model types, predicted flux changes in three metabolites, glutamine, lysine, and lactate, were consistent with alterations observed in the cerebrospinal fluid (CSF) of PD patients. The observed flux changes in glutamine underscore its potential role in coordinating dopaminergic neuron function and contributing to reduced dopamine release. While it is widely recognized that imbalances in glutamine and glutamate can disrupt the glutamine-glutamate-GABA metabolic cycle in glutamatergic neurons, emerging evidence underscores the role of glutamatergic mechanisms in dopaminergic neurons (Plaitakis and Shashidharan, 2000; Eskenazi et al., 2021). Studies have indicated that the co-transmission of dopamine and glutamate may serve as a general mechanism for modulating the activity of dopaminergic and serotonergic cells, thus expanding their range of neurotransmitter actions (Hnasko et al., 2010; Broussard, 2012; Eskenazi et al., 2021). Additionally, the decreased uptake of lysine may reflect compensatory interconversion of other amino acids (Papes et al., 2001), while the reduced uptake of lactate, a key energy substrate, may be linked to the decreased energy activity of dopaminergic neurons in PD condition (Karagiannis et al., 2021).

Consistent flux changes in methionine and urea, which align with those observed in PD CSF metabolites, were predicted exclusively in the synaptic PD model. This finding indicates that these changes may be more specific to the synaptic component. It has been reported that genes regulating methionine concentration in the midbrain are involved in the dopaminergic synaptic signaling pathway, with elevated methionine levels potentially exacerbating neurodegenerative disorders (Wang et al., 2024). Methionine is a widely used, sulfur-containing amino acid involved in protein synthesis, the transsulfuration pathway, and polyamine biosynthesis, such as spermine and spermidine (Parkhitko et al., 2019). Moreover, dopamine-mediated oxidation of methionine 127 in α-Syn can lead to cytotoxicity and oligomerization of α-Syn (Nakaso et al., 2013), a key pathological feature of PD. The flux changes of methionine in the synaptic models may indicate that dysfunctional polyamine metabolism is primarily associated with the synaptic component, which, in turn, could alter ornithine metabolism and affect urea levels via the urea cycle (Luo et al., 2024). Further study is needed to better understand the changes in methionine and urea levels in the synaptic components.

Consistent flux changes in histidine and glyceric acid observed in the non-synaptic PD model may indicate that these alterations are specific to the non-synaptic component of dopaminergic neurons. The depletion of histidine is significantly associated with altered histamine levels, which affect behavioral and electrophysiological responses in humans (van Ruitenbeek et al., 2009). The decreased histidine uptake in the non-synaptic PD model may reflect reduced somal electrophysiological activity in PD condition. However, the role of glyceric acid in dopaminergic neurons remains unclear. Additionally, changes in six metabolites in the CSF of PD patients, including tryptophan, asparagine, isoleucine, ornithine, putrescine, and citric acid, were not observed in either the synaptic or non-synaptic models, as their fluxes reached the upper bound constraint set for dopaminergic neurons. Further investigation is required to elucidate the metabolic connections in the synaptic and non-synaptic components under both control and PD conditions, and to clarify the mechanisms underlying interactions among glial cells, neurons, and CSF.

### 4.3 Bioenergetic rescue target

The single-reaction flux inhibition analysis indicated that inhibition of the mitochondrial phenylalanine transaminase (PHETA1m) reaction could computationally improve the energy state and metabolite exchanges only in the synaptic PD model. This reaction involves two aminotransferases: tyrosine aminotransferase and glutamic-oxaloacetic transaminase 2. Due to their broad substrate specificity, evidence suggests that brain tyrosine aminotransferase and glutamic-oxaloacetic transaminase 2 (GOT2) are functionally identical and play a crucial role in phenylalanine transamination (Benuck, 1980; Mehere et al., 2010; Kerk et al., 2022). In conjunction with observed reaction flux changes, inhibition of PHETA1m appears to trigger a compensatory mechanism that enhances alternative amino acid pathways, as evidenced by increased flux in reactions primarily involved in lysine, isoleucine, and phenylalanine metabolism. This enhanced amino acid metabolism further improved oxidative phosphorylation, particularly via the electron transfer flavoprotein (ETF) coupling pathway. However, validating the involved non-specific enzyme and broadly altered amino acid metabolism remains challenging when the intervention is limited to PHETA1m inhibition.

The analysis of single-reaction flux increasing more effectively identified verifiable targets. The results indicated that an increased flux through the mitochondrial ornithine transaminase reaction (ORNTArm) could enhance bioenergetic performance and metabolite exchanges in both synaptic and non-synaptic PD models. This reaction is catalyzed by the mitochondrial ornithine aminotransferase (OAT, E.C.2.6.1.13), which reversibly transfers the δ-amino group from ornithine to α-ketoglutarate, yielding glutamate-5-semialdehyde and glutamate (Ginguay et al., 2017). It is involved in metabolic pathways connecting glutamate and ornithine to key molecules in the urea cycle, proline metabolism, and polyamine metabolism, with its activity predominantly observed in the intestine, liver, and kidney (Ginguay et al., 2017). In the brain, OAT was found in cortical, hippocampal, and basal ganglia neurons (Kasahara et al., 1986). However, changes in ornithine aminotransferase levels have only been observed in glutamatergic neurons and associated with Huntington’s disease, suggesting a role for OAT in the synthesis of the neurotransmitter glutamate (Wong et al., 1982). In the rescued synaptic and non-synaptic PD models, increased fluxes through the urea cycle following an increase in ORNTArm suggest enhanced ornithine metabolism; this was accompanied by increased oxygen consumption and further improvements in the respiratory chain. Although no evidence has yet established an association between changes in OAT levels and other neurodegenerative disorders, OAT may represent a promising target for alleviating bioenergetic failure in Parkinson’s disease through enhanced ornithine metabolism. Further research could focus on ornithine transaminase to elucidate this mechanism and develop treatment strategies for PD patients.

### 4.4 Limitations and recommendations

Several limitations in this study need to be improved in future research. Although we explored the bioenergetic differences between models within defined constraints under varying energy demands, the mitochondrial activity data derived from rat or mouse mitochondria across various brain regions may limit the interpretation of energy dynamics in the dopaminergic neurons of the human SNpc, particularly under high energy demand. Future experiments are needed to determine *in vivo* mitochondrial enzyme activities of these dopaminergic neuronal components in the human SNpc. Additionally, applying identical uptake constraints across all models, based on exometabolomic data from *in vitro* dopaminergic neurons, limits our ability to capture differences between the control and PD states in synaptic and non-synaptic components. Additional investigation is required to elucidate the metabolic requirements of *in vivo* dopaminergic neurons under both control and PD conditions. Given that predefined constraints (e.g., oxygen uptake) significantly influence model performance, careful consideration of these constraints is essential for different cellular components in future modeling.

Although this study hypothesized that PD CSF metabolites primarily reflect metabolic dysfunction in dopaminergic neurons, interactions among glial cells, neurons, and the CSF may also contribute to these alterations in PD patients. The inconsistency between predicted metabolite exchanges in PD models and observed metabolite changes in the CSF may result from insufficient exploration of these cellular interactions. Future research should focus on metabolic interactions across multiple cells to better elucidate the metabolic changes in the CSF. Given the complex coordination within a neuron, functionally distinguishing between these components remain challenging. Consequently, this study can serve as a reference for further investigations into the distinct roles of synaptic and non-synaptic components. Future research is required to validate the dysfunction mechanisms that contribute to the selective vulnerability of dopaminergic neurons and to explore potential therapeutic strategies for PD patients.

## Conclusion

To explore differences in bioenergetic performance and metabolite exchanges between distinct neuronal components, we generated four genome-scale metabolic models representing the synaptic and non-synaptic components of dopaminergic neurons under both control and PD conditions. In particular, the synaptic PD model showed a significantly reduced ATP contribution from oxidative phosphorylation and increased sensitivity to Complex I inhibition, contributing to the selective vulnerability observed in PD. The consistent and inconsistent metabolite exchanges observed in the synaptic and non-synaptic PD models reflect distinct metabolic patterns between these neuronal components. Additionally, mitochondrial ornithine transaminase was predicted to be the potential bioenergetic rescue target for both the synaptic and non-synaptic PD models. Further research is needed to validate these dysfunctional mechanisms and to explore targeted therapeutic strategies for PD patients.

## Data Availability

The original contributions presented in this study are included in this article/Supplementary material, further inquiries can be directed to the corresponding author.

## Footnotes

1
https://www.vmh.life/

2
https://cellxgene.cziscience.com/
