# Supplementary material for: Constraint-based modeling of bioenergetic differences between synaptic and non-synaptic components of dopaminergic neurons in Parkinson’s disease
Source: Front Comput Neurosci. 2025 Jun 5;19:1594330. doi: 10.3389/fncom.2025.1594330 (PMC12176876; doi:10.3389/fncom.2025.1594330)
Supplement: Supplementary file 1 [file Table_1.docx]

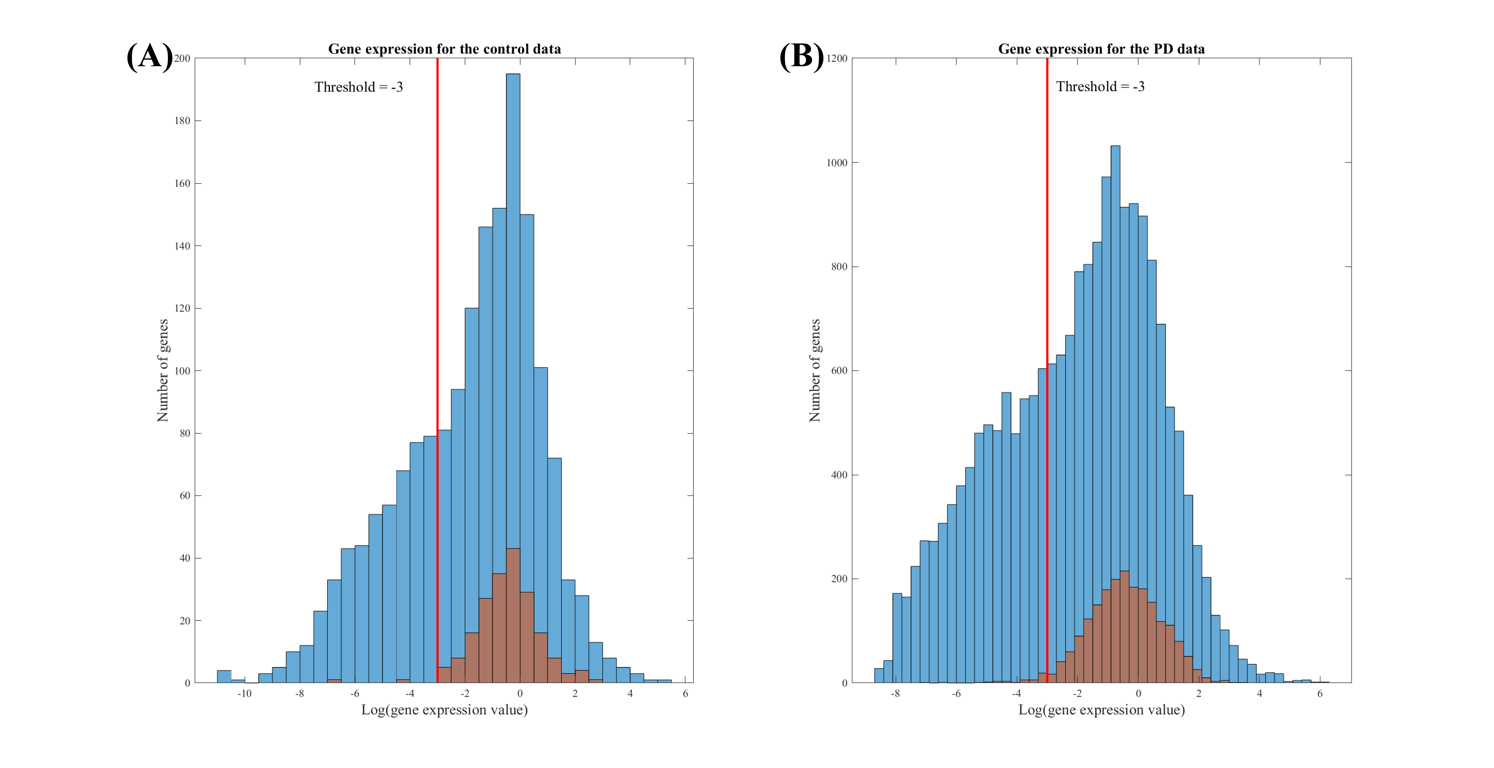


**Supplementary Figure 1.** Bar charts of transcriptomic data for all genes and housekeeping genes

**
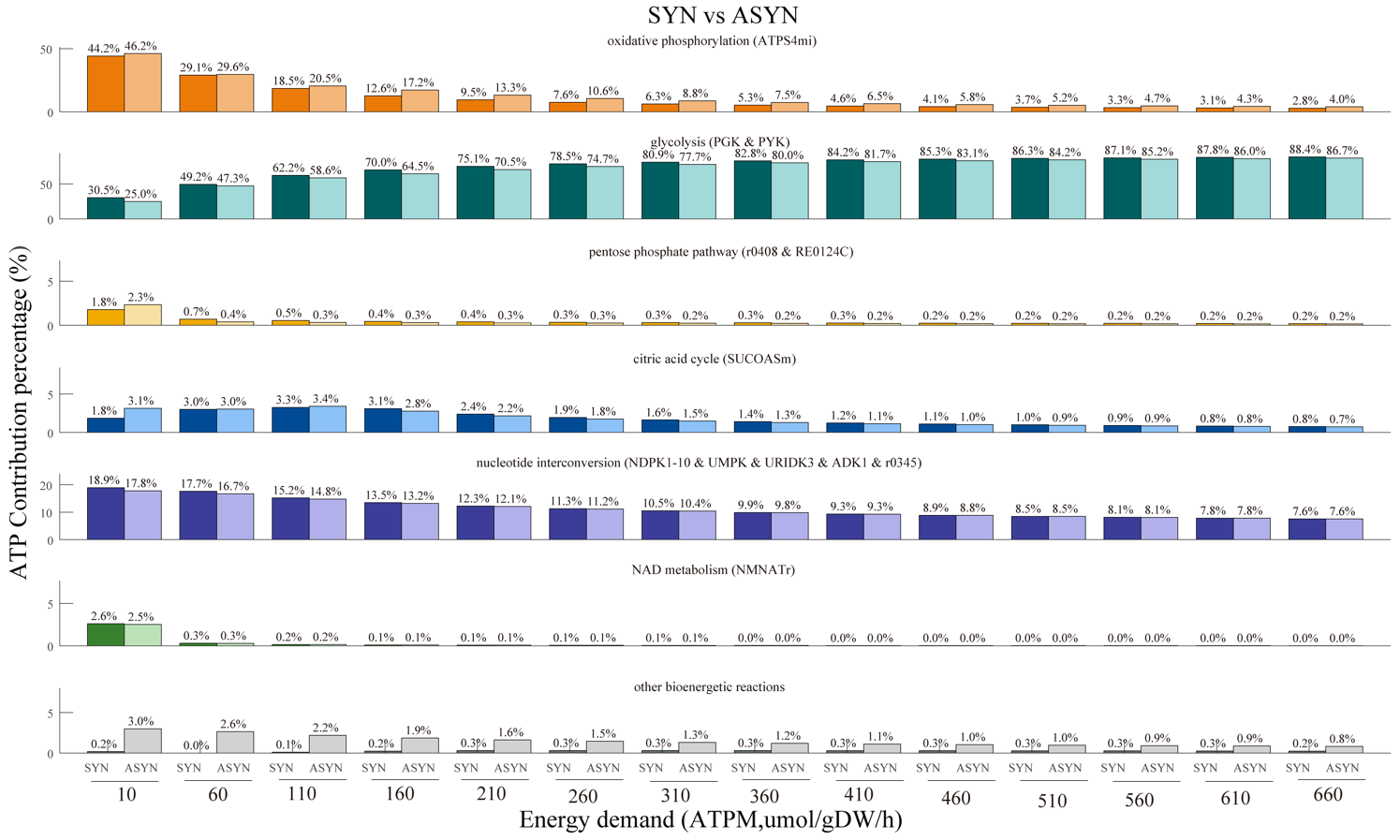
Supplementary Figure 2.** Comparison of ATP contribution between synaptic and non-synaptic models across energy demands from 10 to 600 µmol/gDW/h.


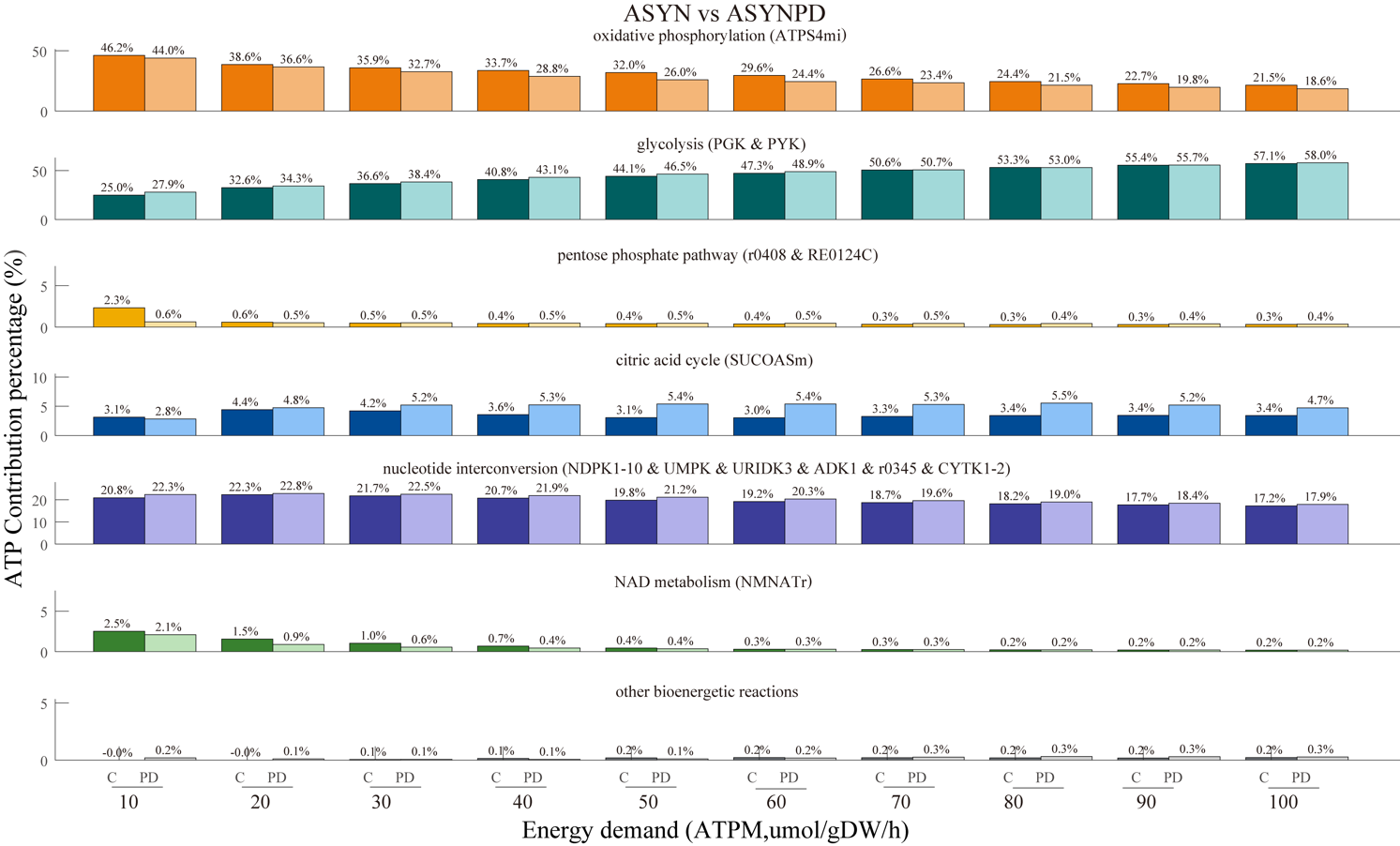


**Supplementary Figure 3.** Comparison of ATP contribution between control and PD non-synaptic models across energy demands from 10 to 100 µmol/gDW/h.


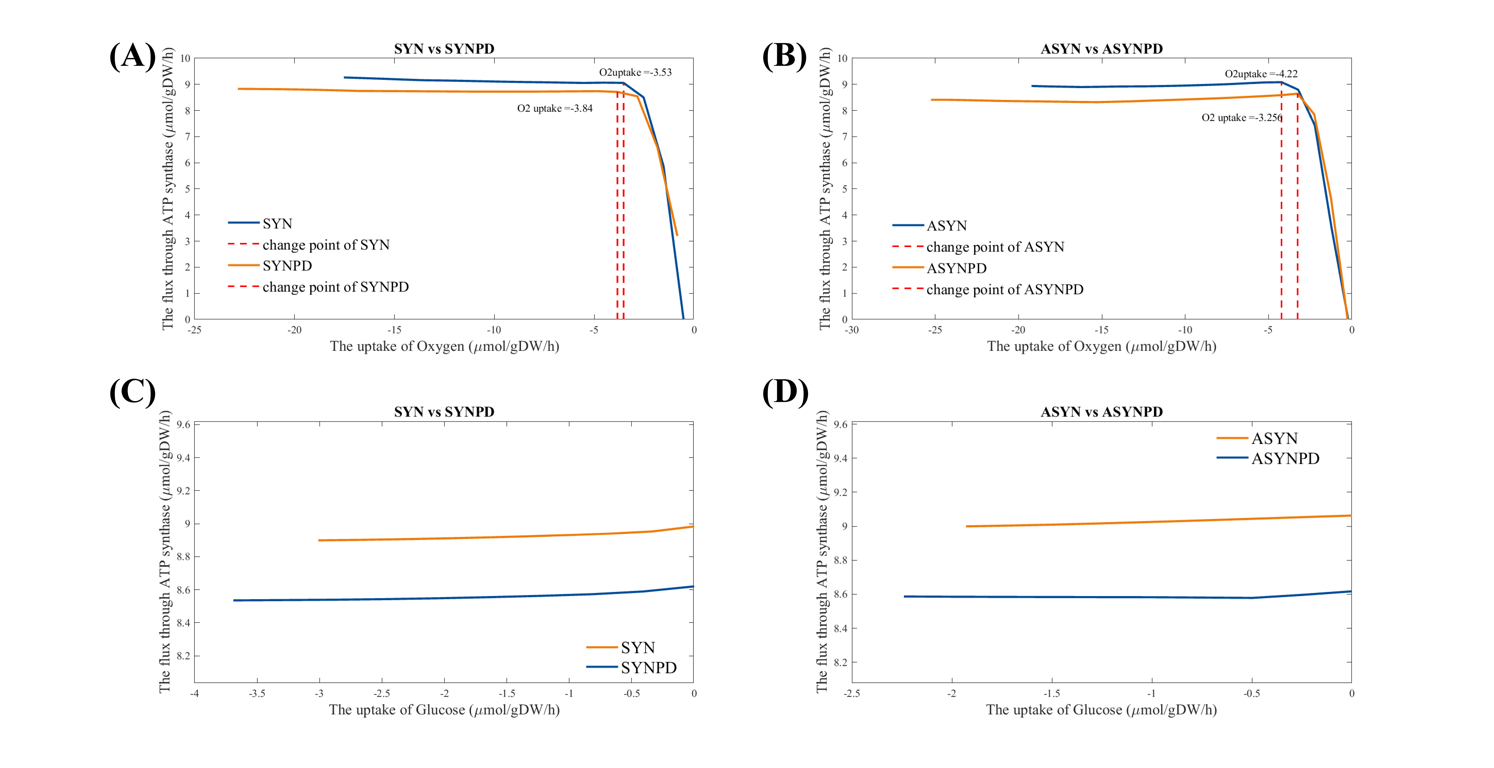


**Supplementary Figure 4.** The sensitivity analysis on oxygen and glucose for the synaptic and non-synaptic models.


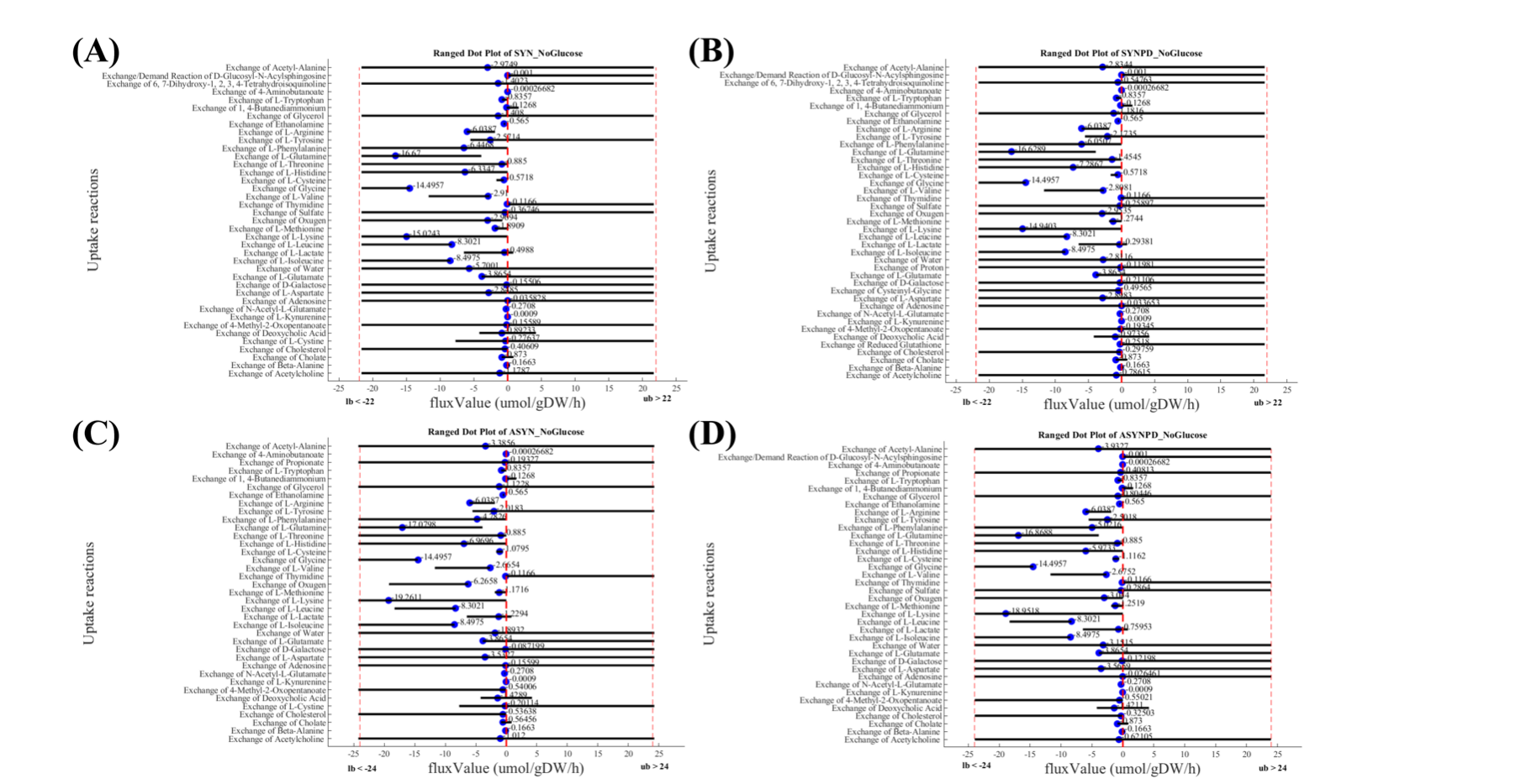


**Supplementary Figure 5.** The uptake reactions in the absence of glucose across all the models.


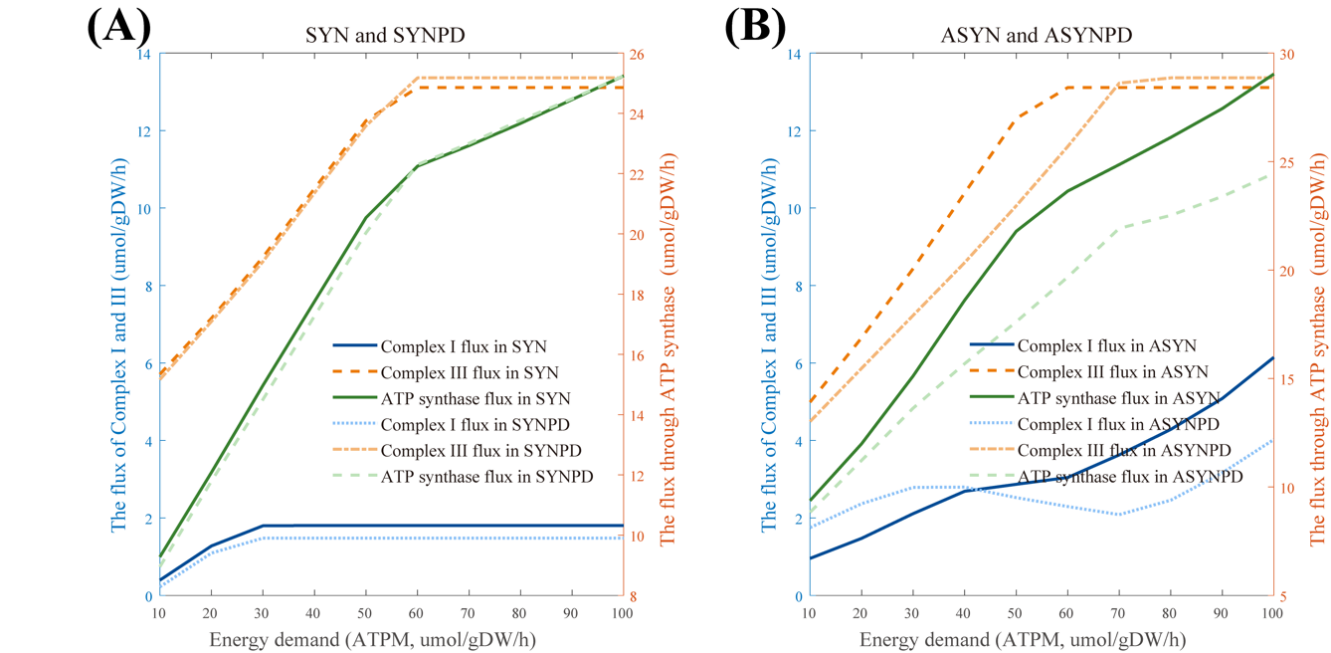


**Supplementary Figure 6.** Flux changes in Complex I, Complex III and ATP synthase under varying energy demands for all the models.


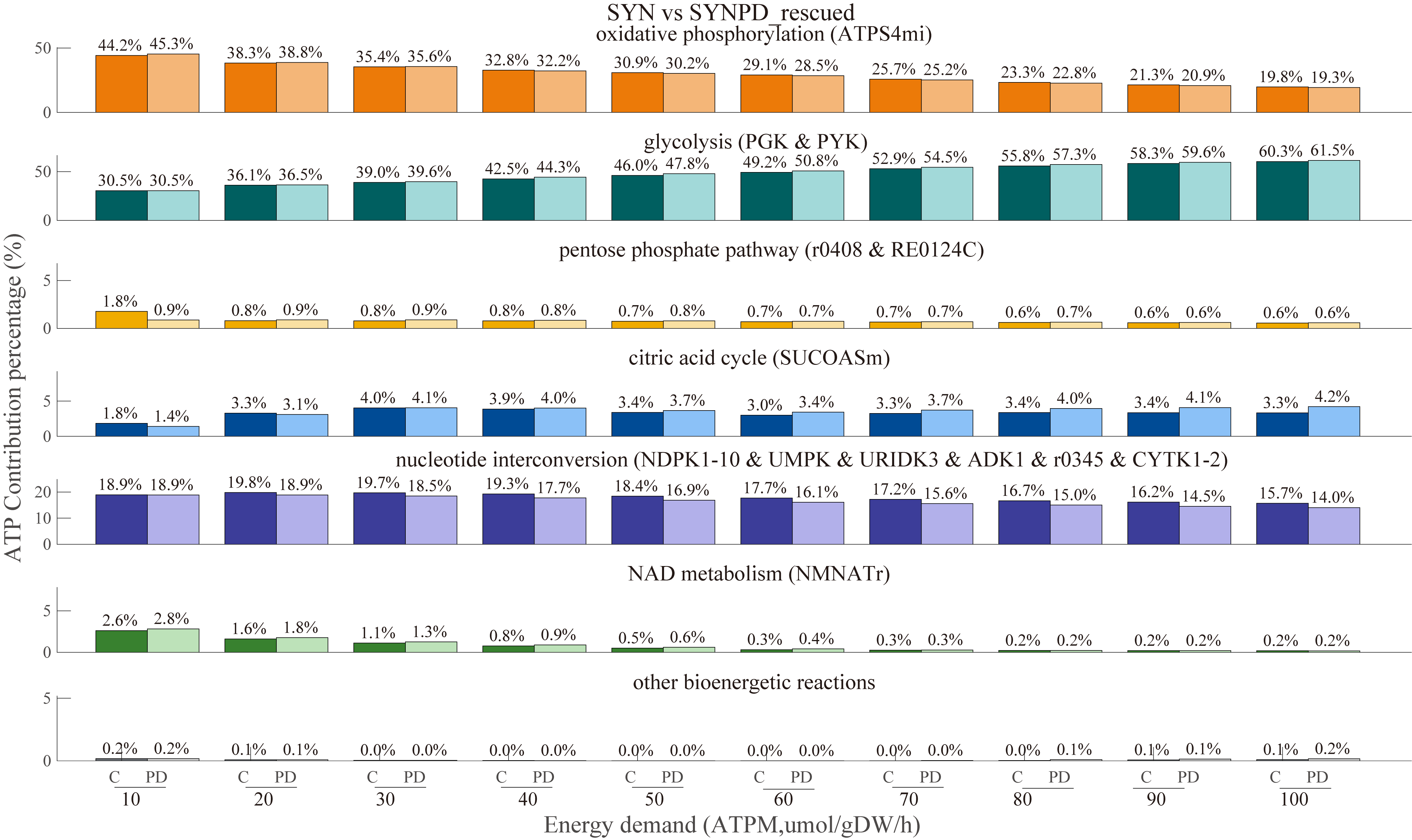


**Supplementary Figure 7.** Comparison of ATP contribution between the synaptic control and rescued PD models across energy demands from 10 to 100 µmol/gDW/h.


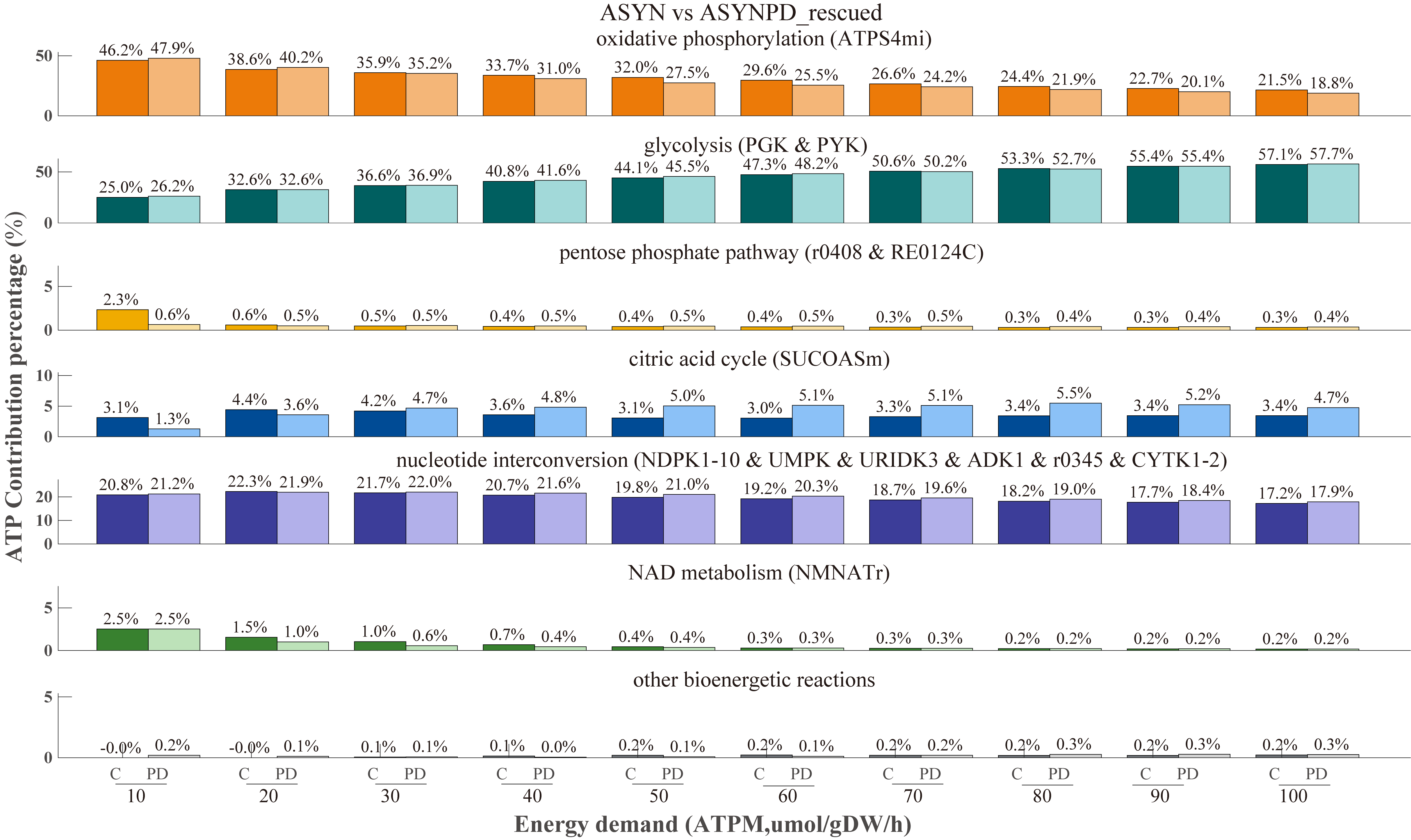


**Supplementary Figure 8.** Comparison of ATP contribution between the non-synaptic control and rescued PD models across energy demands from 10 to 100 µmol/gDW/h.
